# Prognostic models for mortality and hospitalisation risk in a contemporary Australian chronic obstructive pulmonary disease cohort

**DOI:** 10.1186/s12931-026-03531-7

**Published:** 2026-02-05

**Authors:** Luke A. Smith, Minyan Zeng, Alix Bird, Anand Rose, Sutapa Mukherjee, Lyle J. Palmer

**Affiliations:** 1 Australian Institute for Machine Learning, University of Adelaide, Adelaide, South Australia Australia; 2 School of Public Health, University of Adelaide, Adelaide, South Australia Australia; 3 Department of Respiratory and Sleep Medicine, Flinders Medical Centre, Adelaide, South Australia Australia; 4 College of Medicine and Public Health, Flinders University, Adelaide, South Australia Australia

**Keywords:** ‘Pulmonary disease, Chronic obstructive’, Prognosis, Regression, Exacerbation, Mortality

## Abstract

**Background:**

Chronic Obstructive Pulmonary Disease (COPD) poses significant public health and economic challenges and performant prognostic models may be useful to direct treatment. The purpose of this study was to develop predictive models, validate the DOSE and updated ADO predictive models, and identify predictors of future hospitalisation and mortality in contemporary Australian COPD patients.

**Methods:**

Data from 8,578 inpatients and outpatients diagnosed with COPD (via post-bronchodilator spirometry) between 2006 and 2021 at a large South Australian tertiary public hospital were analysed. Multivariate logistic models and Cox regression, utilising penalised regularisation in multiply imputed data, were used to investigate predictors of hospitalisation due to COPD exacerbation at 1-, 3-, and 5-years post-diagnosis, and COPD-specific mortality at 3- and 5-years. Haemoglobin-corrected DLCO (DLCOc) was used to extend the DOSE and updated ADO models.

**Results:**

Locally developed models could predict COPD-specific 1-year hospitalisation risk with AUCs 0.80 (95% CI = [0.76, 0.83]) in males and 0.82 (95% CI = [0.78, 0.86]) in females on a temporally distinct hold-out set, with 3- and 5-year AUCs falling within this range. COPD-specific mortality was predicted with AUCs of 0.90 (95% CI = [0.85, 0.94]) and 0.89 (95% CI = [0.84, 0.92]) at 3 and 5 years in females, and 0.90 (95% CI = [0.86, 0.93]) and 0.88 (95% CI = [0.84, 0.92]) in males. Cox regression models predicted survival well in the test set for both females (C-index = 0.88, 95% CI = [0.85, 0.90]) and males (C-index = 0.86, 95% CI = [0.82, 0.88]). Local model performance was superior to that of the DOSE and updated ADO models for all outcomes, although not always significantly. Among the selected predictors, reduced DLCOc was strongly predictive of all outcomes. and acts as a short-term survival risk for follow-up durations less than 10 years. There was no significant difference in performance between sex, and there were differences in selected features and feature strength between sexes. Extending the extant clinical models with DLCOc significantly improved updated ADO and DOSE model fit and improved discriminatory performance, with the extended ADO index achieving AUC of 0.77 (95% CI = [0.75, 0.79]) and 0.87 (95% CI = [0.84, 0.89]) for predicting 5-year hospitalisation and mortality respectively across the full cohort. The extended DOSE index performed similarly with AUCs 0.77 (95% CI = [0.75, 0.79]) and 0.87 (95% CI = 0.83, 0.89)) for 5-year hospitalisation and mortality.

**Conclusions:**

Ours is the only large clinical cohort and prognostic study of Australian COPD patients to date. Locally developed models achieved greater discriminative performance than the original updated ADO and DOSE models within our cohort. Extending the ADO and DOSE models with DLCOc significantly improved model fit in our cohort. We recommend further research into the use of DLCOc as a prognostic index for COPD, and its inclusion in future modelling attempts.

**Trial registration:**

Retrospectively registered. Clinical trial number: Not Applicable.

**Supplementary Information:**

The online version contains supplementary material available at 10.1186/s12931-026-03531-7.

## Background

Chronic Obstructive Pulmonary Disease (COPD) is a heterogeneous disease characterised by chronic airflow obstruction and persistent respiratory symptoms [1]. The major risk factor for COPD is tobacco smoking, but other environmental factors (*e.g.* passive exposure to ambient pollution, occupational particulate matter, or second-hand tobacco smoke) and genetic factors (primarily alpha-1 antitrypsin deficiency [2]) also play important roles in disease aetiology [1]. In spite of best efforts at prevention, COPD remains prevalent with a high disease burden, contributing to 5.4% of Australian deaths and 5.8% of global deaths in 2019 [3]. COPD is among the top 5 disorders that contribute to disability-adjusted life years (DALY) lost in Australia [4]. In excess of 53,000 patients over the age of 45 were hospitalised in Australia with a principal diagnosis of COPD in 2020–2021, and it is estimated COPD cost the Australian health system almost $995 million dollars in 2019–2020 [5]. In the US, estimated direct medical costs for COPD were $46.91 billion USD in 2019, estimated to increase to $57.86 billion annually by 2038 [6]. There is an unmet need for precision approaches to better inform treatment priorities and decisions in COPD [7]. In patients with COPD, disease heterogeneity confounds our abilities to appropriately prescribe suitable treatments until after the disease has progressed [8, 9].

A problem with current clinical approaches is that those with severe COPD receive the most aggressive treatments only after they have already experienced repeated exacerbations, which themselves can cause irreversible reductions in lung function as well as increase the risk of future exacerbations and mortality risk [8, 10]. Clinically useful prognostic models to identify patients at risk of adverse outcomes are hence of great clinical interest [11]. There have been over 400 publications investigating such prognostic models to date. However, a recent meta-analysis suggested that most suffer from poor methodological rigor and are not generalizable [11]. A few, such as the “Body-mass index, airflow Obstruction, Dyspnoea, and Exercise capacity” (BODE) index [12], the “Age, Dyspnoea, and airflow Obstruction” (ADO) index [13], and the “Dyspnoea, airflow Obstruction, Smoking Status, and Exacerbation frequency” (DOSE) index [14], have been externally validated with methodological rigor, although meta-analysis of their pooled discriminatory performance during external validation yielded modest results (Area under receiver operator characteristic curve (AUC) ranged between 0.61–0.77) [11]. More recent models incorporating machine learning and imaging features have experienced similar problems and offer no significant improvement in discriminatory ability [15]. Thus far, prognostic indices have seen limited clinical use as a consideration before surgical interventions and during follow-up after hospitalisation [9, 16]. Aside from the goal to improve clinical practice, prognostic models are also of scientific interest as they may identify prognostic factors related to disease aetiology and allow better phenotyping [17].

Australia has a national public health insurance scheme covering 100% of its population, with 54.9% of the population also holding private health insurance as of 2022 [18]. The pharmaceutical benefits scheme provides subsidies to approved medication which includes long-acting beta-agonists (LABA), long-acting muscarinic antagonists (LAMA), and inhaled corticosteroids (ICS), enabling all patients with COPD affordable access to medication. Population level research in South Australia is supported at both the state-level by SA-NT Datalink, and nationally by the Population Health Research Network, enabling data linkage to span the full breadth of the Australian public health system. Demographically, South Australia represents an ageing population with 19% of its population aged over 65 in 2020, though the median age is slightly lower than the United States of America and the United Kingdom [19]. Neither the updated ADO nor the DOSE metrics were developed or updated on Australian cohorts, and due to differences in clinical practice and cohort demographics, performance and behaviour of these models may not translate well to an Australian setting.

Our study aimed to use rigorous statistical approaches to create prognostic models for key COPD clinical outcomes using data from a large, retrospective cohort of South Australian COPD patients to provide a baseline for prognostic performance within an Australian context. We then aimed to validate the DOSE and ADO indices on our cohort to compare performance in the original development cohorts and our local models. Finally, we extended the DOSE and ADO models in an attempt to improve their performance without the South Australian setting and thus approach the baseline set by the locally developed models.

## Methods

### Study population

A total of 8,578 patients visited the three lung function laboratories (Flinders Medical Centre, Noarlunga GP Plus, and the Repatriation General Hospital) of the Southern Adelaide Local Health Network (SAHLN) in Adelaide Australia between 1 st January 2006 and 31 st December 2021. Patients were eligible for inclusion in our study if they received a diagnosis of COPD, defined by a post-bronchodilator FEV_1_/FVC ratio < 0.7, as recorded in the SALHN lung function testing (SentrySuite, Vyaire, version 3.2.0) database.

Patients younger than 45 or older than 90 years at diagnosis were excluded. No patients were excluded based on missing data, except for those without a post-bronchodilator FEV_1_/FVC. Data for this study were collected with a waiver of consent approved by the SAC HREC (LNR/22/SAC/2, Clinical trial number: not applicable).

The routinely collected clinical and spirometric data available on patients included:

1. Demographic characteristics: Age and biological sex [M | F].
2. Physical characteristics: Height, weight, and body mass index (BMI).
3. Smoking status [never | ex | current].
4. Mortality status, date of death, and mortality diagnostic codes.
5. Diagnostic details and dates of COPD-related hospitalisation events.
6. Spirometric Measurements: Forced expiratory volumes after 1 s (FEV_1_), forced vital capacity (FVC), their ratio (FEV_1_/FVC), and the ratio between forced expiratory volume at 1 and 6 s (FEV_1_/FEV_6_); forced inspiratory vital capacity (FIVC); peak expiratory flow (PEF); and maximal mid-expiratory flow (MMEF – the ratio of the difference between lung volume at 75% and 25% inflation against the time difference between these two lung volumes during forced expiration).
7. Other Clinical Measurements: Saturation of peripheral oxygen (SpO_2_), Diffusing Capacity of Lung for Carbon Monoxide corrected for Haemoglobin (DLCOc), and modified Medical Research Council questionnaire score (mMRC) [20].

Demographic and spirometric data were measured at the SALHN lung function laboratories by trained respiratory scientists during routine visits. Accurate ancestry/ethnic status was not available.

COPD-specific hospitalisation and COPD-specific mortality data were acquired through linkage to state-wide administrative data from all public hospitals in SA, approved by the DHW HREC (2023/HRE00024). A hospitalisation or mortality event was deemed as COPD-specific if it received a primary or secondary ICD-10 diagnosis code of J44.0 (COPD with acute lower respiratory infection), J44.1 (COPD with acute exacerbation, unspecified), or J44.9 (COPD, unspecified) [21]. Hospitalisation and mortality data from 1 st Jan 2000 to 31 st July 2024 was linked.

### Statistical analyses

All statistical analyses were performed using the R software package v4.5.1 [22].

Baseline function/visit was defined as the first visit by patients to the lung function laboratory with a diagnosis of COPD via spirometry.

Sex, and hospitalisation due to COPD exacerbation in the past year were encoded as binary variables (female = 0, male = 1; no-hospitalisation = 0, hospitalisation = 1). Normally distributed continuous variables had outliers removed. SpO_2_ was normalised through a log_e_-transformation before analysis. Prior to multivariate analysis, all continuous predictive variables were mean centred and divided by twice the standard deviation to put them on approximately the same scale as the binary variables, per Gelman’s recommendations [23].

Smoking status was recorded as a categorical variable (never-smoker = 0, ex-smoker = 1, or current smoker = 2). The mMRC score was recorded as a categorical variable (range 0 to 4). Before modelling, smoking status and mMRC were encoded using 2 and 4 dummy variables respectively, following the split-coding contrast scheme [24]. Regression coefficients for the dummy variables correspond to differences between consecutive levels.

The two binary outcomes assessed were hospitalisation due to COPD exacerbation at 1, 3, and 5 years post baseline, and COPD-specific mortality at 3, and 5 years. There was insufficient outcome data for 1-year COPD-specific mortality to be modelled. Hospitalisation events at each time point were recorded as true if there had been at least one hospitalisation coded with a primary or secondary diagnosis of COPD since baseline.

For each outcome time period (1, 3, 5 years), we removed all patients whose first visit did not have sufficient follow-up time and then divided the datasets into temporally distinct train-test splits where the final 5 years of the dataset time period were put into the test set. This resulted in differently sized datasets for each follow-up time.

To account for missing data, multivariate imputation by chained equations (MICE) [25] was used to uniquely generate 40 imputed datasets under the assumption that data were missing at random [26]. Stability of imputations was observed after 15 iterations. The training and testing datasets were imputed separately to avoid leakage of test data into the training sets.

Multivariate logistic regression models were fit to each binary outcome, stratified by sex. Prior work has shown that sex modifies several key measures used in COPD including lung volumes, and morbidity, and susceptibility to smoking [27, 28], and our exploratory testing of sex-predictor interactions supported this evidence. Stratified modelling allowed us to capture these differences without assuming uniform effects across predictors and provided flexibility for sex-specific predictors to be selected.

To select a set of predictors across imputed datasets to include in the final model and mitigate the effects of overfitting, we used an elastic net penalty function [29], the Stacking adaptive Elastic NET (SaENET) algorithm, as implemented in the MISELECT package [30]. Regularisation techniques such as elastic net penalty functions reduce risk of bias to overfitting, reduce complexity in modelling, and increase parsimony between models [29, 31]. Final models were chosen such that the degree of regularisation was maximised while keeping the fivefold cross validation error within one standard deviation of the minimum cross-validation error.

We evaluated the models on the train and test datasets, reporting AUCs, sensitivity, and specificity along with 95% Confidence Intervals (95% CIs) pooled across the imputed datasets using Rubin’s Rules [32]. We selected the ‘top-left’ corner of the ROC curve as the operating point for measuring sensitivity and specificity. Calibration intercept, referred to as ‘Calibration In the Large’ (CIL) or mean calibration [33], and calibration slope were calculated by fitting the log-odds linear predictors to the outcomes using a binomial GLM. Estimates for intercept (CIL) and slope were pooled using Rubin’s Rule. P-values testing the null hypotheses that intercept was 0 and slope was 1 were generated and the median across imputations reported, where *p <* 0.05 indicates significant miscalibration in the CIL or slope. Calibration curves were created by aggregating the predicted probabilities for patient data into 10 deciles, and plotting mean observed event proportion to mean predicted probability of each decile. A spline with three knots was used to draw the curve.

The DOSE [14] and updated ADO [34] indices were calculated and validated in our cohort. The BODE [12] index could not be calculated as this index requires six-minute walk distances, which were not available for over 95% of our cohort. To assess model fairness across sex, we compared model test set AUCs between sex using DeLong’s test [35] and reported the median p-values across imputations. We compared our local models’ discriminative capacity against the DOSE and updated ADO by comparing AUCs with DeLong’s test.

We then extended and refit the DOSE and updated ADO indices on our full (unstratified) training sets, incorporating DLCOc as an additional predictor based on early results highlighting its consistent predictive power for all outcomes. Improvement in model fit was assessed using D_2_ test statistic implemented in MICE [25]. Extended indices were evaluated on the full test set for each outcome, as well as the sex-stratified subsets, and performance between the extended indices and the original indices were compared using DeLong’s test.

In addition to predicting mortality at each time point, we assessed time-dependent risk of mortality as a censored outcome by fitting multivariate Cox Proportional Hazards models on the female and male cohorts, using only data available at baseline to predict hazard. Data imputation was performed using the methods described above. Because the MISELECT package has no implementation for Cox regression models, we used the glmnet package [29, 36] in R to implement stacked elastic-net regression without the adaptive weights available in MISELECT. We evaluated prognostic performance using the C-index measured on a randomly held out test set of 20% of the baseline cohort after fitting to 80%. Additionally, after fitting to the training set, we used the hazard model to assign a score to each patient in the test set. We then split the scores into high-low groups at the median hazard score and drew Kaplan–Meier curves for each sex split into high-low risk, and reported the Hazard Ratios for sex, high-low risk, and the interaction between the two.

All p-values reported were adjusted for repeated testing using the Bonferroni-Holm correction to correct for type-I error [37].

## Results

Characteristics of the study population at the baseline visit are summarized in Table 1. Continuous variables were reported as mean and standard deviation. Categorical and binary variables had the count and percentage for each category reported. Descriptive statistics were reported on the full cohort and for males and females individually.

**Table 1:** Characteristics of study population: total population, females, and males at time of first assessment

Variable	All (*n* = 8,578)	Female (*n* = 3,727)	Male (*n* = 4,851)
Age (years)^a^	69.86 (10.36)	69.33 (10.53)	70.26 (10.21)*
Height (m)^a^	167.16 (9.45)	159.73 (6.58)	172.87 (7.04)*
Weight (kg)^a^	79.95 (20.05)	73.19 (19.31)	85.15 (19.04)*
BMI (kg/m^2^)^a^	28.51 (6.41)	28.64 (7.18)	28.41 (5.74)
FEV_1_ (L)^a^	1.87 (0.75)	1.52 (0.54)	2.14 (0.77)*
FVC (L)^a^	3.31 (1.06)	2.62 (0.70)	3.84 (0.98)*
FIVC (L)^a^	2.99 (1.02)	2.38 (0.69)	3.47 (0.99)*
FEV_1_/FVC (%)^a^	56.30 (11.98)	57.50 (11.27)	55.38 (12.42)*
FEV_1_/FEV_6_ (%)^a^	63.31 (9.98)	63.87 (9.39)	62.89 (10.39)*
PEF (L/s)^a^	5.67 (2.17)	4.51 (1.49)	6.56 (2.18)*
MMEF (L/s)^a^	0.78 (0.46)	0.65 (0.36)	0.88 (0.51)*
DLCOc (ml/min/kPa)^a^	5.30 (2.02)	4.58 (1.56)	5.81 (2.15)*
SpO_2_ (%)^a^	96.11 (2.20)	96.05 (2.15)	96.15 (2.24)
Hospitalisation (Past Year)^b^	975 (11.4%)	455 (12.2%)	520 (10.7%)
Smoking Status^b^	8,096 (94.1%)	3,456 (92.7%)	4,613 (95.1%)*
Never	1,131 (13.2%)	675 (18.1%)	456 (9.4%)
Ex	4,894 (57.1%)	1,830 (49.1%)	3,064 (63.2%)
Current	2,044 (23.8%)	951 (25.5%)	1,093 (22.5%)
mMRC^b^	7,508 (87.5%)	3,295 (88.4%)	4,213 (86.8%)*
0	1,562 (18.2%)	484 (13.0%)	1,078 (22.2%)
1	2,769 (32.3%)	1,293 (34.7%)	1,476 (30.4%)
2	1,514 (17.6%)	749 (20.1%)	765 (15.8%)
3	922 (10.7%)	407 (10.9%)	515 (10.6%)
4	741 (8.6%)	362 (9.7%)	379 (7.8%)
Hospitalisation^b,c^			
1 year	1,228 (14.3%)	582 (15.6%)	646 (13.3%)*
3 years	1,982 (23.1%)	918 (24.6%)	1,064 (21.9%)*
5 years	2,380 (27.7%)	1,101 (29.5%)	1,279 (26.4%)*
Mortality^b,d^			
1 year	79 (0.9%)	26 (0.7%)	53 (1.1%)
3 years	296 (3.5%)	123 (3.3%)	173 (3.6%)
5 years	501 (5.8%)	208 (5.6%)	293 (6.0%)

*BMI* Body Mass Index, *DLCOc* Diffusing capacity of the Lung for carbon monoxide (CO) corrected for haemoglobin, *FEV*_*1*_ Forced Expiratory Volume in 1 s, *FEV*_*6*_ Forced Expiratory Volume in 6 s, *FIVC* Forced Inspiratory Vital Capacity, *FVC* Forced Vital Capacity, *MMEF* Maximal Mid-Expiratory Flow, *mMRC* modified Medical Research Council Questionnaire Score, *PEF* Peak Expiratory Flow, *SpO*_*2*_ Saturation of Peripheral Oxygen

^a^Mean (SD)

^b^n (%)

^c^COPD-related hospitalisation events with admission or separation ICD-10 codes of J44.0, J44.1, or J44.9 within *n* years of initial presentation

^d^All-cause mortality event within *n* years of initial presentation

We report variables where there was a significant difference in distribution between males and females with ‘*’. Continuous variables were compared using a two-sided t-test, categorical/binary variables were compared using chi-squared test

Mean age was 69.9 years (SD = 10.4) and 4,851 (56.6%) of the patients were male. Mean FEV_1_ was 1.87 L (SD = 0.75), and mean FEV_1_/FVC ratio was 56.3% (SD = 12.0). 6,938 patients (80.9%) were ever smokers, with 4,894 (57.1%) ex-smokers and 2,044 (23.8%) current smokers. Males had a significantly higher rate of ex-smokers than females (63.2% vs 49.1%). In the year preceding baseline spirometry 975 (11.4%) of patients had been hospitalised with an admission code corresponding to COPD diagnosis; 1,228 (14.3%) were hospitalised in the year following baseline. There was a significant difference between male and female sex in all variables assessed at baseline except BMI and SpO_2_. Within 5 years post baseline, 2,380 (27.7%) of patients had been hospitalised due to COPD at least once, and 501 (5.8%) of patients had died as a result of COPD. COPD-specific hospitalisation rate was higher in females than males but there was no significant difference between proportions of COPD-specific mortality. A total of 1,002 (11.7%) patients ever experienced COPD-specific mortality with a median survival time of 59 months (Interquartile Range (IQR) = [30, 96]). For patients without COPD-specific mortality, median censoring time was 88 months (IQR = [46, 144]).

Table S1 in supplementary material describes the percentage of missing data for each variable. FIVC had the most missing entries at 31.3% missing, SpO_2_ 26.0%, DLCOc 13.9%, mMRC 12.5%, smoking status 6.1%, and FEV_1_/FEV_6_ and MMEF were both missing less than 1%. Table S2 in supplementary material reports the number of outcome events and patients in the training and testing datasets for each outcome stratified by sex. Female 3-year mortality had the fewest events in the training and testing datasets with 96 (3.8%) and 26 (2.4%) respectively, while male 5-year hospitalisation had the most events, 760 (27.9%) and 386 (26.7%) respectively making it the most prevalent outcome.

### Logistic models predicting mortality and hospitalisation

Table 2 shows the odds ratios from sex-stratified regularised logistic regression models for each binary outcome. Across both sexes, MMEF (protective), DLCOc (protective), Hospitalisation (past year) (risk factor), and mMRC (risk factor) were retained for all outcomes, though SpO_2_ and smoking status were also retained for all outcomes in females. Between 9 (male 1-year hospitalisation) and 15 (female 5-year hospitalisation) unique predictors were selected for each model. The association of prior hospitalisation was strongest when predicting future hospitalisation versus predicting mortality.

**Table 2:** Odds ratios from hospitalisation and mortality prognostic models in females and males

Variable	**Female**					**Male**				
	**Hosp.**^a^** 1 years**	**Hosp.**^a^** 3 years**	**Hosp.**^a^** 5 years**	**Mort.**^**b**^** 3 years**	**Mort.**^**b**^** 5 years**	**Hosp.**^a^** 1 years**	**Hosp.**^a^** 3 years**	**Hosp.**^a^** 5 years**	**Mort.**^**b**^** 3 years**	**Mort.**^**b**^** 5 years**
Time^c^	1.453	1.497	2.253	-	-	1.247	1.502	1.903	-	-
Age^c^	-	-	-	-	-	-	-	-	1.041	1.077
Age^2,c^	-	-	0.847	-	-	-	-	0.989	-	-
Height	-	-	-	-	-	-	-	-	-	-
Weight	-	-	-	-	-	-	-	-	0.998	-
BMI	-	-	1.008	-	-	-	-	-	0.964	0.978
FEV_1_	0.811	0.830	0.827	0.679	0.716	-	-	-	0.855	-
FVC	-	-	-	0.953	0.876	-	-	-	0.916	-
FIVC	-	-	-	-	-	-	-	-	-	-
FEV_1_/FVC	-	-	0.999	0.998	0.990	-	0.992	0.984	0.997	0.989
FEV_1_/FEV_6_	0.978	0.978	0.968	-	-	0.980	0.978	0.981	-	-
PEF	-	-	-	0.875	0.851	0.893	0.910	0.926	0.954	-
MMEF	0.640	0.611	0.787	0.849	0.879	0.768	0.837	0.748	0.914	0.747
DLCOc	0.867	0.820	0.805	0.807	0.784	0.911	0.847	0.882	0.804	0.722
SpO_2_^d^	1.330	1.353	1.415	1.650	1.402	-	1.270	1.331	1.546	1.662
Hospitalisation (Past Year)	2.453	2.402	2.867	2.314	1.954	2.585	2.776	2.648	1.785	1.868
mMRC										
0	Reference	Reference	Reference	Reference	Reference	Reference	Reference	Reference	Reference	Reference
1	1.063	1.123	1.245	-	-	1.256	1.315	1.317	1.160	-
2	1.212	1.357	1.796	-	-	1.463	1.373	1.317	1.532	1.287
3	1.354	1.469	2.056	1.561	1.678	1.463	1.373	1.317	2.154	1.310
4	1.354	1.469	2.056	1.790	1.678	1.715	1.373	1.317	2.154	1.524
Smoking Status										-
Never	Reference	Reference	Reference	Reference	Reference	Reference	Reference	Reference	Reference	Reference
Ex	1.189	1.153	1.451	-	-	-	1.137	1.220	-	-
Current	1.189	1.153	1.828	1.006	1.158	-	1.160	1.425	-	-

*BMI* Body Mass Index, *DLCOc* Diffusing capacity of Lung for Carbon Monoxide corrected for haemoglobin, *FEV*_*1*_ Forced Expiratory Volume in 1 s, *FEV*_*6*_ Forced Expiratory Volume in 6 s, *FIVC* Forced Inspiratory Vital Capacity, *FVC* Forced Vital Capacity, *mMRC* modified Medical Research Council Questionnaire Score, *SpO*_*2*_ Saturation of Peripheral Oxygen

^a^COPD-specific hospitalisation events with admission or separation ICD-10 codes of J44.0, J44.1, or J44.9 within *n* years of initial presentation

^b^COPD-specific mortality event within *n* years of initial presentation

^c^Reported as effect per decade, not per year

^d^SpO_2_ has been transformed using the following function: f(SpO_2_) = ln(101- SpO_2_) where ln is the natural logarithm and SpO_2_ is originally reported as a percentage from 0 to 100. Odds ratio is with respect to the transformed variable

Tables 3 and 4 report the model performances on the training and test sets respectively. Hospitalisation events were predicted with AUCs between 0.79–0.81 on the training set and 0.79–0.82 on the testing set. COPD-specific mortality events were predicted with AUCs between 0.87–0.89 on the training set and 0.88–0.90 on the testing set. There was no notable performance degradation when moving from training to testing set, suggesting the elastic net regularisation has been successful in limiting overfitting, at least across a temporally distinct holdout set. Elastic net fivefold cross validation error curves and parameter penalisation curves are provided in the supplementary materials (figures S3-12). Pooled confidence intervals are larger in the test set than the training set, partially due to fewer outcome events.

**Table 3:** Discriminative performance of penalised logistic models for hospitalisation and mortality in females and males (training dataset)

Outcome	**Female**			**Male**		
	**AUC (95% CI)**	**Sensitivity** **(95% CI)**	**Specificity** **(95% CI)**	**AUC (95% CI)**	**Sensitivity** **(95% CI)**	**Specificity** **(95% CI)**
Hosp.^a^ 1 year	0.793 (0.771, 0.814)	0.731 (0.656, 0.794)	0.719 (0.642, 0.785)	0.797 (0.776, 0.816)	0.737 (0.666, 0.797)	0.715 (0.657, 0.766)
Hosp.^a^ 3 years	0.804 (0.785, 0.822)	0.741 (0.693, 0.783)	0.723 (0.675, 0.765)	0.809 (0.792, 0.826)	0.729 (0.684, 0.769)	0.739 (0.693, 0.781)
Hosp.^a^ 5 years	0.805 (0.784, 0.825)	0.757 (0.702, 0.806)	0.707 (0.653, 0.756)	0.797 (0.778, 0.814)	0.722 (0.666, 0.772)	0.723 (0.666, 0.774)
Mort.^b^ 3 years	0.887 (0.851, 0.915)	0.825 (0.711, 0.900)	0.814 (0.721, 0.881)	0.892 (0.863, 0.915)	0.814 (0.718, 0.883)	0.813 (0.733, 0.874)
Mort.^b^ 5 years	0.870 (0.837, 0.897)	0.801 (0.706, 0.871)	0.778 (0.696, 0.844)	0.876 (0.851, 0.898)	0.821 (0.730, 0.887)	0.779 (0.710, 0.836)

*95% CI* 95% Confidence Interval, *AUC* Area under receiver-operating characteristic curve

^a^COPD-specific hospitalisation events with admission or separation ICD-10 codes of J44.0, J44.1, or J44.9 within *n* years of initial presentation

^b^COPD-specific mortality event within *n* years of initial presentation

**Table 4:** Discriminative performance of penalised logistic models for hospitalisation and mortality in females and males (testing dataset)

Outcome	**Female**			**Male**			
	**AUC (95% CI)**	**Sensitivity (95% CI)**	**Specificity (95% CI)**	**AUC (95% CI)**	**Sensitivity (95% CI)**	**Specificity (95% CI)**	**Adjusted p-values**
Hosp.^a^ 1 year	0.822 (0.778, 0.860)	0.758 (0.656, 0.837)	0.756 (0.640, 0.844)	0.797 (0.756, 0.833)	0.731 (0.638, 0.808)	0.729 (0.625, 0.813)	p = 1.0
Hosp.^a^ 3 years	0.809 (0.775, 0.839)	0.728 (0.639, 0.801)	0.759 (0.665, 0.833)	0.803 (0.773, 0.830)	0.690 (0.626, 0.747)	0.774 (0.694, 0.838)	p = 1.0
Hosp.^a^ 5 years	0.817 (0.789, 0.842)	0.740 (0.677, 0.794)	0.741 (0.673, 0.798)	0.803 (0.777, 0.827)	0.726 (0.671, 0.775)	0.732 (0.669, 0.786)	p = 1.0
Mort.^b^ 3 years	0.901 (0.846, 0.938)	0.834 (0.575, 0.949)	0.759 (0.639, 0.849)	0.902 (0.859, 0.933)	0.838 (0.688, 0.924)	0.827 (0.685, 0.913)	p = 1.0
Mort.^b^ 5 years	0.887 (0.843, 0.919)	0.842 (0.700, 0.924)	0.790 (0.712, 0.851)	0.880 (0.841, 0.910)	0.783 (0.662, 0.869)	0.826 (0.688, 0.911)	p = 1.0

*95% CI* 95% Confidence Interval, *AUC* Area under receiver-operating characteristic curve

We compared the performance of our models between males and females using two-sided DeLong’s test on each imputed test set and report the median p-values. Median-pooled p-values were adjusted using the Bonferroni-Holm correction

^a^COPD-related hospitalisation events with admission or separation ICD-10 codes of J44.0, J44.1, or J44.9 within *n* years of initial presentation

^b^COPD-specific mortality event within *n* years of initial presentation

Although the selected covariates were different in males and females, there was no statistically significant difference in model predictive performance between males and females in the test datasets (adjusted median p-values = 1.0), suggesting the model does not advantage one sex over another.

**Fig. 1:**
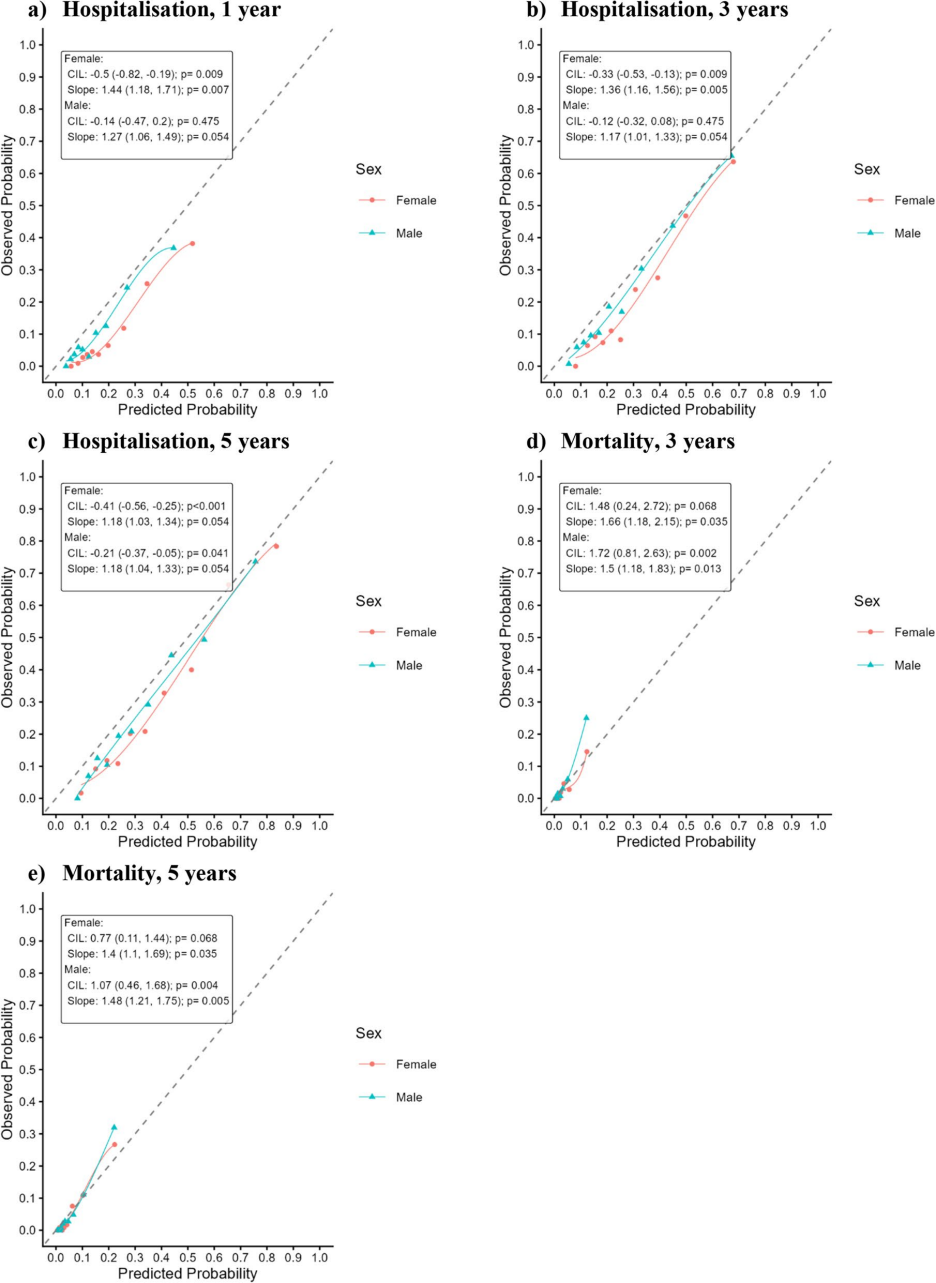
Pooled calibration plots for hospitalisation and mortality prognostic models in males and females. Pooled calibration curves, stratified by sex, for prediction of COPD-related hospitalisation events after 1 (**a**), 3 (**b**), and 5 years (**c**) and COPD-specific mortality events after 3 (**d**) and 5 years (**e**). Predicted probabilities were grouped by decile, and calibration curve drawn with a loess function. The dashed diagonal line represents perfect calibration

Model calibration is reported in Table 5. Based on CIL and calibration slope metrics, only the models predicting 1- and 3-year hospitalisation in males did not have significant miscalibration on both the intercept and slope. Calibration slope for the 5-year hospitalisation model for males and females did not deviate significantly from unity, and CIL for 3- and 5-year mortality models for females did not deviate significantly from 0. For males and females, the hospitalisation risk prognostic models exhibited slight systemic overestimation of risk (CIL < 0), while mortality risk prognostic models exhibited systemic underestimation of risk (CIL > 0) [33]. Estimated calibration slopes for all models were greater than 1, indicating predicted risks were too conservative. Low risk patients have even lower risk than predicted, while high risk patients have higher risk than predicted [33]. Visual inspection of calibration curves in Fig. 1 confirms moderate risk overestimation in hospitalisation models. The mortality prediction models were well calibrated in the < 10% probability range, but deviated from the diagonal for risk $\ge$ 10%, underestimating the true proportion of mortality events.

**Table 5:** Calibration of penalised logistic regression prognostic models for hospitalisation and mortality in males and females (testing cohort)

Outcome	**Female**		**Male**	
	**CIL (95% CI); *p*-value**	**Calibration Slope (95% CI); *p*-value**	**CIL (95% CI); *p*-value**	**Calibration Slope (95% CI); *p*-value**
Hosp.^a^ 1 year	−0.504 (−0.818, −0.189); *p* = 0.009	1.443 (1.179, 1.707); *p* = 0.007	−0.136 (−0.469, 0.197); *p* = 0.475	1.271 (1.056, 1.487); *p* = 0.054
Hosp.^a^ 3 years	−0.330 (−0.534, −0.127); *p* = 0.009	1.359 (1.157, 1.561); *p* = 0.005	−0.120 (−0.319, 0.079); *p* = 0.475	1.172 (1.014, 1.331); *p* = 0.054
Hosp.^a^ 5 years	−0.405 (−0.559, −0.251); *p <* 0.001	1.182 (1.028, 1.335); *p* = 0.054	−0.210 (−0.366, −0.054); *p* = 0.041	1.183 (1.036, 1.330); *p* = 0.054
Mort.^b^ 3 years	1.480 (0.237, 2.723); *p* = 0.068	1.662 (1.178, 2.146); *p* = 0.035	1.716 (0.805, 2.626); *p* = 0.002	1.505 (1.182, 1.828); *p* = 0.013
Mort.^b^ 5 years	0.772 (0.108, 1.437); *p* = 0.068	1.399 (1.104, 1.693); *p* = 0.035	1.068 (0.460, 1.676); *p* = 0.004	1.478 (1.208, 1.749); *p* = 0.005

*95% CI* 95% Confidence Interval, *CIL* Calibration In the Large

For CIL, p > 0.05 means there is insufficient evidence to suggest the model calibration intercept deviates from 0, *i.e.* there is no systemic over- or underestimation. CIL > 0 implies systemic underestimation of risk, while CIL < 0 implies systemic overestimation of risk. For Calibration slope, p > 0.05 means there is insufficient evidence to suggest that model calibration slope deviates from 1, *i.e.* estimated risks are not too conservative or too extreme. Calibration slope > 1 implies estimated risk were too conservative. *i.e.* estimates for high-risk patients were too low and estimates for low-risk patients were too high. Slope < 1 implies estimated risks were too extreme, *i.e.* estimates for high-risk patients were too high and estimates for low-risk patients were too low

^a^COPD-related hospitalisation events with admission or separation ICD-10 codes of J44.0, J44.1, or J44.9 within *n* years of initial presentation

^b^COPD-specific mortality event within *n* years of initial presentation

The updated ADO index could predict hospitalisation with AUCs between 0.72 and 0.77, and mortalities with AUCs between 0.82 and 0.86. The DOSE index could predict hospitalisation with AUCs between 0.69 and 0.77, and mortalities with AUCs between 0.78 and 0.84. In all cases, point estimates for our model AUC exceeded the updated ADO and DOSE models evaluated on the test set (Table 6). However, when comparing against the updated ADO, the difference was only significant among females for hospitalisation events (adjusted *p <* 0.05), while our performance was only significantly better than the DOSE index for female 5-year hospitalisation, and male 3- and 5-year hospitalisation and mortality. We note that the DOSE index generally performed better on females within our cohort than males, while the updated ADO index performed better in males, though the differences across sex were not significant.

**Table 6:** Comparison of performance between established predictive indices and the current study’s models for hospitalisation and mortality on test data

Outcome	Ours	Updated ADO^c^	DOSE^d^
*Female*	**AUC (95% CI)**	**AUC (95% CI)**	**AUC (95% CI)**
Hospitalisation^a^ 1 year	0.822 (0.778, 0.860)	0.718 (0.667, 0.763); p = 0.014	**0.772 (0.719, 0.817); p = 0.792**
Hospitalisation^a^ 3 years	0.809 (0.775, 0.839)	0.730 (0.692, 0.765); p = 0.017	**0.746 (0.705, 0.783); p = 0.137**
Hospitalisation^a^ 5 years	0.817 (0.789, 0.842)	0.740 (0.709, 0.769); p = 0.002	0.714 (0.680, 0.747); *p <* 0.001
Mortality^b^ 3 years	0.901 (0.846, 0.938)	**0.829 (0.746, 0.889); p = 0.732**	**0.840 (0.719, 0.915); p = 1.0**
Mortality^b^ 5 years	0.887 (0.843, 0.919)	**0.823 (0.773, 0.864); p = 0.301**	**0.808 (0.740, 0.862); p = 0.284**
*Male*			
Hospitalisation^a^ 1 years	0.797 (0.756, 0.833)	**0.745 (0.701, 0.785); p = 0.665**	**0.749 (0.702, 0.791); p = 0.792**
Hospitalisation^a^ 3 years	0.803 (0.773, 0.830)	**0.767 (0.735, 0.795); p = 0.716**	0.722 (0.686, 0.755); p = 0.004
Hospitalisation^a^ 5 years	0.803 (0.777, 0.827)	**0.754 (0.726, 0.780); p = 0.091**	0.687 (0.655, 0.717); *p <* 0.001
Mortality^b^ 3 years	0.902 (0.859, 0.933)	**0.858 (0.806, 0.898); p = 0.948**	0.784 (0.700, 0.849); p = 0.048
Mortality^b^ 5 years	0.880 (0.841, 0.910)	**0.854 (0.814, 0.887); p = 1.00**	0.784 (0.723, 0.835); p = 0.042

*95% CI* 95% Confidence Interval, *AUC* Area under receiver-operating characteristic curve

We compared the performance of our models and established predictive indices performed using DeLong’s test, with alternative hypothesis that our model performance was greater, on each imputed test set and report the median p-values. Median p-values were adjusted using Bonferroni-Holm correction. We have marked the p-values where our model was not significantly better on the test set (p > = 0.05) in bold

^a^COPD-related hospitalisation events with admission or separation ICD-10 codes of J44.0, J44.1, or J44.9 within *n* years of initial presentation

^b^COPD-specific mortality event within *n* years of initial presentation

^c^Age, Dyspnoea, Airflow Obstruction index [34]

^d^Dyspnoea, Airflow Obstruction, Smoking, Exacerbation index [14]

The Wald D_2_ test showed significant improvement in model fit across all outcomes for the updated ADO and DOSE models after adding DLCOc (Table S3). Extended model performances were calculated on the testing set and reported in Table 7, and extended coefficients are provided in supplementary materials Table S3. Incorporation of DLCOc improves point-estimate AUC for all outcomes, sexes, and models when compared to the original indices, with DOSE benefiting the most from the update, though in females the difference is only significant for 5-year hospitalisation risk (ADO adjusted p = 0.019, DLCO adjusted *p <* 0.001). The improvement in updated ADO due to DLCOc is only significant for 1-year hospitalisation in males, and 1- and 5-year hospitalisation for the full cohort (males and females combined). The DOSE index significantly benefits from DLCOc in males and the full cohort in all outcomes.

**Table 7:** Model performance for updated ADO and DOSE indices extended to include DLCOc

Outcome	Updated ADO^c^ + DLCOc^e^	DOSE^d^ + DLCOc^e^
*Female*	**AUC (95% CI)**	**AUC (95% CI)**
Hospitalisation^a^ (1 year)	**0.752, (0.698, 0.799); *p =* 0.104**	**0.800, (0.751, 0.841); *p =* 0.187**
Hospitalisation^a^ (3 years)	**0.747, (0.707, 0.783); *p =* 0.472**	**0.769, (0.729, 0.804); *p =* 0.363**
Hospitalisation^a^ (5 years)	0.770, (0.739, 0.798); *p =* 0.019	0.779, (0.748, 0.807); *p <* 0.001
Mortality^b^ (3 years)	**0.881, (0.787, 0.936); *p =* 0.472**	**0.891, (0.801, 0.943); *p =* 0.753**
Mortality^b^ (5 years)	**0.851, (0.791, 0.895); *p =* 0.753**	**0.854, (0.790, 0.900); *p =* 0.457**
*Male*		
Hospitalisation^a^ (1 year)	0.785, (0.744, 0.821); *p =* 0.024	0.809, (0.771, 0.843); *p =* 0.001
Hospitalisation^a^ (3 years)	**0.776, (0.743, 0.806); *p =* 0.898**	0.780, (0.748, 0.809); *p =* 0.002
Hospitalisation^a^ (5 years)	**0.774, (0.746, 0.800); *p =* 0.275**	0.768, (0.739, 0.794); *p <* 0.001
Mortality^b^ (3 years)	**0.889, (0.834, 0.927); *p =* 0.480**	0.890, (0.833, 0.929); *p =* 0.012
Mortality^b^ (5 years)	**0.887, (0.848, 0.918); *p =* 0.201**	0.885, (0.844, 0.917); *p <* 0.001
*All*		
Hospitalisation^a^ (1 year)	0.766, (0.734, 0.795); *p =* 0.004	0.797, (0.766, 0.824); *p <* 0.001
Hospitalisation^a^ (3 years)	**0.758, (0.733, 0.782); *p =* 0.753**	0.767, (0.742, 0.790); *p =* 0.003
Hospitalisation^a^ (5 years)	0.770, (0.749, 0.789); *p =* 0.012	0.769, (0.748, 0.788); *p <* 0.001
Mortality^b^ (3 years)	**0.877, (0.833, 0.911); *p =* 0.434**	0.879, (0.833, 0.914); *p =* 0.012
Mortality^b^ (5 years)	**0.867, (0.835, 0.893); *p =* 0.275**	0.866, (0.832, 0.893); *p <* 0.001

*95% CI* 95% Confidence Interval, *AUC* Area under receiver-operating characteristic curve

We compared the performance of the extended ADO and DOSE models against the original ADO and DOSE models using DeLong’s test, with alternative hypothesis that extended model performance was greater, on each imputed test set and report the median p-values. Median p-values were adjusted using Bonferroni-Holm correction. We have marked the p-values where the extended model was not significantly better than the existing model on the test set (p > = 0.05) in bold

^a^COPD-specific hospitalisation events with admission or separation ICD-10 codes of J44.0, J44.1, or J44.9 within *n* years of initial presentation

^b^COPD-specific mortality event within *n* years of initial presentation

^c^Age, Dyspnoea, Airflow Obstruction index [34]

^d^Dyspnoea, Airflow Obstruction, Smoking, Exacerbation index [14]

^e^Diffusing capacity of the Lungs for Carbon Monoxide Corrected for haemoglobin

A cohort effect (date of diagnosis relative to 2006) was positively associated with hospitalisation in males and females, increasing at later years. By comparison, age and its squared term were infrequently selected. Height was never selected, weight was only selected for 3-year mortality in males, and BMI for 5-year hospitalisation in females, and 3- and 5-year mortality in males. It is likely these sparsely selected variables are more a result of statistical phenomena which provided minor improvements to model fit than meaningful results.

Lung-function indices showed sex-specific patterns. Higher FEV_1_ was protective against all outcomes for females, but only 3-year mortality risk for males. FVC was protective against mortality for females, and against 3-year mortality in males. FIVC was never selected. FEV_1_/FVC and FEV_1_/FEV_6_ were broadly protective in both sexes and though based on inclusion frequencies, there is evidence to suggest FEV_1_/FVC is a better indicator of mortality while FEV_1_/FEV_6_ is more associated with hospitalisation risk. Lower PEF was predictive of mortality in females, and all outcomes except 5-year hospitalisation in males. Lower oxygen saturation (higher log(101-SpO_2_)) predicted hospitalisation and mortality in both sexes except for 1-year hospitalisation in males.

Dyspnoea (mMRC) was a consistent predictive factor for hospitalisation and mortality in both sexes. In females, increasing mMRC levels corresponded to stepwise increases in hospitalisation risk up to grade 3, with grade 4 collapsing to grade 3. Mortality risk only increased for mMRC $\ge$ 3. In males, mMRC > 0 collapsed to a single risk for 3- and 5-year hospitalisation, but incremental differences remained for 1-year hospitalisation. 3- and 5-year mortality both showed incrementing risk at higher mMRC grades.

Smoking status was only weakly predictive of 3- and 5-year mortality for female current smokers but was generally predictive of hospitalisation for both males and females. For females, there is only a difference in risk between ex and current smokers when predicting 5-year hospitalisation risk whereas male current smokers were at higher risk than ex-smokers at 3- and 5-years.

Formulae for all predictive models are provided in the Supplementary Material.

### Cox Proportional Hazards survival models for COPD-specific mortality

Cox Proportional Hazards models with elastic net regularisation were trained on 2,953 females and 3,909 males, with 343 (11.6%) and 454 (11.6%) experiencing COPD-specific mortality. In the 20% random hold-out test set, 97/774 (12.5%) females and 101/942 (10.7%) males experienced COPD-specific mortality. Table 8 reports the hazard ratios for the Cox regression models post stacked elastic-net regularisation. Among males and females age, lower FEV_1_, lower FEV_1_/FVC, lower FEV_1_/FEV_6_, lower PEF, lower MMEF, lower oxygen saturation, and a hospitalisation in the past year were all predictive of mortality. Increasing dyspnoea (i.e., mMRC levels) corresponded to stepwise increases in risk for males and females, although mMRC = 1 was indistinguishable from mMRC = 0 among females. Smoking status was only a significant hazard among current smoker females. Additionally, lower FIVC was predictive of mortality in females, and lower BMI and weight were predictive in males, though the effect size for weight is very small. Height and FVC were never selected.

**Table 8:** Hazard ratios for multivariate Cox regression

Variable	Female	Male
Age^a^	1.080	1.149
Age^2,a^	-	-
Height	-	-
Weight	-	1.000
BMI	-	0.990
FEV_1_	0.641	0.753
FVC	-	-
FIVC	0.937	-
FEV_1_/FVC	0.996	0.992
FEV_1_/FEV_6_	0.987	0.992
PEF	0.869	0.976
MMEF	0.770	0.896
DLCOc (0–5 years)	0.808	0.705
DLCOc (5–10 years)	0.853	0.835
DLCOc (10–15 years)	-	-
DLCOc (15 + years)	-	-
SpO_2_^b^	1.096	1.107
Hospitalisation (Past Year)	1.773	1.579
mMRC = 0	Reference	Reference
mMRC = 1	-	1.015
mMRC = 2	1.048	1.262
mMRC = 3	1.496	1.526
mMRC = 4	1.566	1.667
Smoking Status = Never	Reference	Reference
Smoking Status = Ex	-	-
Smoking Status = Current	1.194	-

*DLCOc* Diffusing capacity of Lungs for Carbon Monoxide corrected for haemoglobin, *FEV*_*1*_ Forced Expiratory Volume in 1 s, *FVC* Forced Vital Capacity, *MMEF* Maximal Mid-Expiratory Flow, *mMRC* modified Medical Research Council Questionnaire Score

^a^Reported as effect per decade, not per year

^b^SpO_2_ has been transformed using the following function: f(SpO_2_) = ln(101- SpO_2_) where ln is the natural logarithm and SpO_2_ is originally reported as a percentage from 0 to 100. Hazard ratio is with respect to the transformed variable

DLCO corrected for haemoglobin strongly violated the proportional hazards assumption of the Cox models (adjusted *p <* 0.001) and needed to have different coefficients selected at different time intervals. Decreased DLCOc was predictive of mortality during the first 5 years, less predictive between 5–10 years post-baseline, and was not predictive of mortality events after 10 years.

Table 9 presents the predictive prognostic performance of the Cox regression models for each sex in the training and testing cohort. Because the Cox models utilised time-stratified coefficients for DLCOc, linear predictors during the first 5 years were used to calculate the c-indices across the full follow-up period. On the training data, females and males achieved similar c-indices of 0.85 (95% CI = [0.83, 0.87]) and 0.86 (95% CI = [0.84, 0.87]). There was little change in performance on the testing set except for widening confidence intervals, with female c-index being 0.88 (95% CI = [0.85, 0.90]) male c-index being 0.86 (95% CI = [0.82, 0.88]).

**Table 9:** Model performance for Cox regression models

Sex	Training C-Index (95% CI)	Testing C-index (95% CI)
Female	0.853 (0.833, 0.870)	0.875 (0.845, 0.899)
Male	0.857 (0.841, 0.872)	0.855 (0.821, 0.884)

95% CI = 95% Confidence Interval

**Fig. 2:**
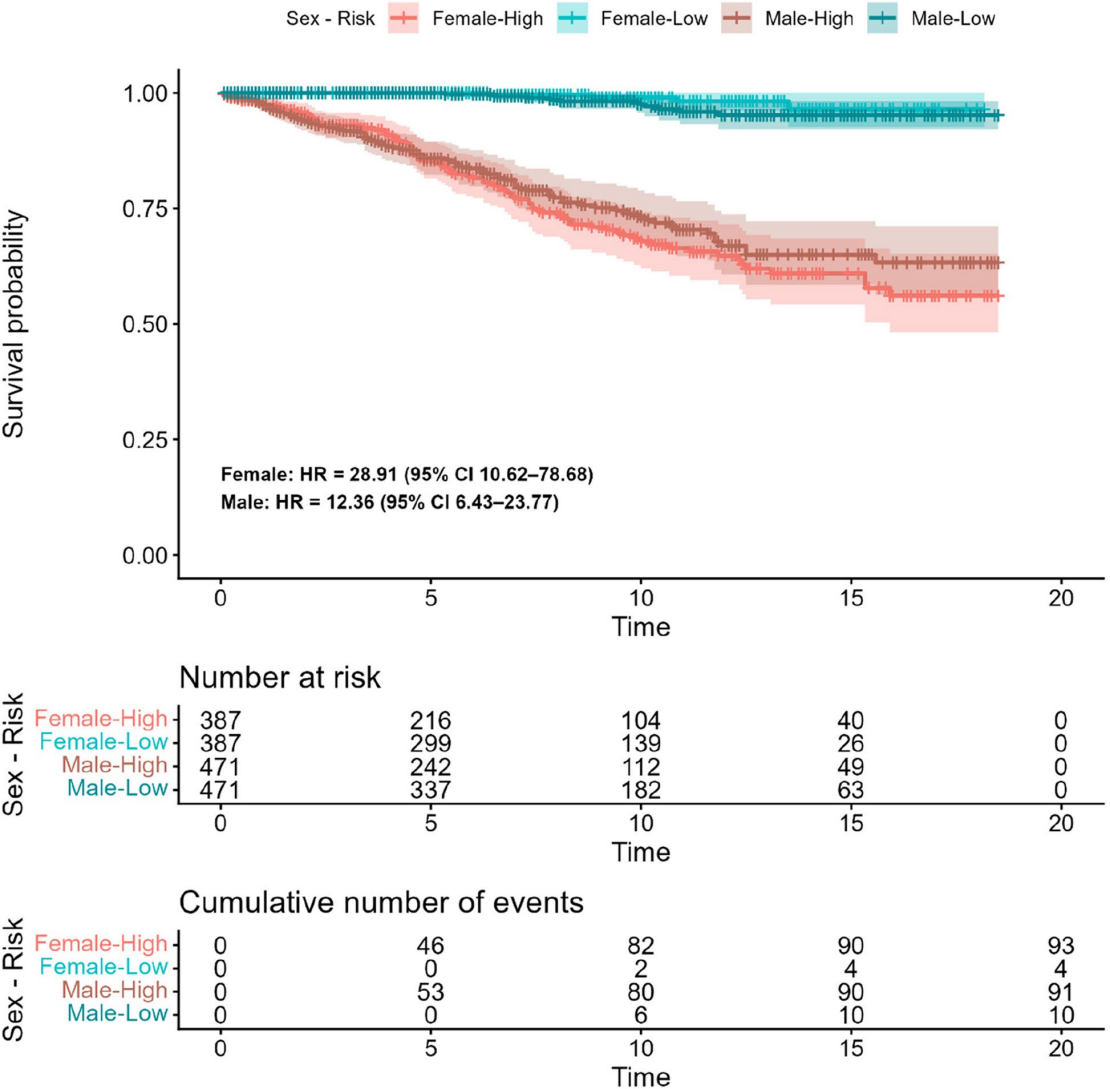
Kaplan–Meier curve for survival stratified by sex and median hazard

Using the fitted Cox models, we assigned a hazard score to each patient in the test set. High- and Low-risk groups were created for males and females by splitting at the median score, and corresponding Kaplan–Meier curves were generated for each Sex-Risk pair, presented in Fig. 2. The hazard ratio for the high-risk classification was 28.9 (95% CI = [10.62–78.68]) among females and 12.4 (95% CI = [6.43, 23.77]) among males.

## Discussion

We have investigated predictive models for COPD outcomes in a large and well characterised contemporary cohort reflecting modern clinical practice in Australia. Our cohort comprised all patients diagnosed with COPD in a major Australian tertiary hospital network over a 14-year period. To the best of our knowledge, the current study represents the only large Australian COPD clinical cohort reported to date.

We developed multivariate prognostic models from multiply imputed data using stacked adaptive elastic nets to investigate the demographic and clinical predictors of risk of hospitalisation and mortality. The discriminatory power of our prognostic models was on par with other well-developed prognostic models for mortality or acute exacerbation [11, 38], and on par with widely used clinical risk scores used for cardiac surgery patients [39], all of which have AUC between 0.7–0.8. Feature selection through LASSO regularisation did not reveal any major trends in sex-specific predictors.

Model discriminative performance was the priority of these modelling efforts, within the constraints of well-informed, parsimonious modelling. There was insufficient outcome data to fit data-hungry forest-based models which benefit from over 200 events per variable (EPV) during training. Even regularisation was not strong enough for 3-year mortality within our local models, with EPV = 8.7 in females and 8.0 in males, putting those models at high risk of bias [40], suggesting that the observed test-set AUC of 0.90 in males and females is likely inflated by overfitting. Hospitalisation for both sexes at all time points, however, was at low risk of bias due to overfitting, a claim which is empirically supported by the negligible differences between training and testing performance.

Calibration metrics also suggested overfitting for the 3-year mortality models with wider confidence intervals in CIL and calibration slope for 3-year mortality in males and females. Although the CIL p-value in females for 3- and 5-year mortality risk did not suggest miscalibration (adjusted *p* = 0.068), this was likely a result of imprecision in the estimated CIL rather than robust calibration.

Although discriminative performance was high, possibly due to poor model calibration, we do not recommend these models be used to provide probabilities of events to patients. Model recalibration would be essential before translation to a clinical setting.

Within the collected cohort DLCOc, FIVC, and SpO_2_ suffer from substantial data missingness of 13.8%, 31.3% and 26.0% respectively. Multiple imputation provides a less biased modelling approach than complete case analysis, even when there are high rates of missingness [41]. Sensitivity analysis of imputed chains and variable distributions reveals no obvious instability or bias in imputed values (Supplementary Figure S1 and S2). Though it is hard to test for, it is reasonable to assume data are missing at random as major disease severity status, such as FEV1/FVC, hospitalisation history, and hospitalisation/mortality outcomes, were all included during imputation.

We validated updated ADO and DOSE models on our test set, obtaining similar or higher AUCs than those reported in pooled analysis of c-statistics by Bellou [11] who reported updated ADO’s c-statistic for mortality to be 0.70 (95% CI = [0.62, 0.76]) and DOSE’s c-statistic for acute exacerbation of COPD and all-cause mortality at 0.61 (95% CI = [0.29, 0.86]) and 0.62 (95% CI = [0.55, 0.69]), suggesting that hospitalisation and mortality prognosis was easier within our cohort than in the developmental cohorts. While the COPD-specific hospitalisation (a proxy for severe acute exacerbation of COPD) AUC for DOSE fell within this range on our test set for males and females at all time points, both the updated ADO and DOSE index reported substantially higher estimates for mortality in both sexes at all time points. One major difference with our cohort was the use of COPD-specific mortality, instead of all-cause mortality which is more common, but confounded by factors not related to COPD. Another point of difference is the comparatively low event count in our dataset for mortality, particularly in the 3-year testing set which has 26 and 50 events in females and males respectively, which could bias the model towards optimistic performance. Events per variable for updated ADO and DOSE are greater than 10 when considering hospitalisation and 5-year mortality in both sexes, and provide more reliable estimates of model performance within our dataset. This holds true for the locally developed models and the model extensions for the updated ADO and DOSE.

After simple extension of the updated ADO and DOSE models incorporating haemoglobin-corrected DLCO, discriminatory performance increased substantially, accounting for almost all the difference between our models and the extant model performance. The DOSE model benefited most from updating, however, this is to be expected as the DOSE uses hard cut-points to create an integer metric which can be used quickly and easily by clinicians to make risk assessments, whereas the ADO model provides a continuous score which needs to be calculated, preferably via digital software. The significance of the D_2_ test for including DLCOc as an additional predictor indicates that DLCO_c_ accounts for a degree of disease status which is not captured by age, dyspnoea, forced expiratory volumes, smoking status, or exacerbation history.

The ADO and DOSE models have undergone extensive validation on multiple large datasets involving tens of thousands of patients and have been assessed as being at low risk of bias. Given the overabundance of prognostic models for COPD [11], the relative ease of model updating and extension, and the incorporation of clinically tested information, we recommend that in circumstances where prognostic models are needed for a local health context, that one of the existing ADO or DOSE models, or BODE where possible, are deployed, possibly with a minor but parsimonious recalibration update or the addition of a feature of interest. The improvement gained from complex models incorporating multiple features does not outweigh the risks of bias introduced during model development or the increased complexity in data generating methods.

The subset of variables found to be predictive of mortality and hospitalization in our contemporary cohort is largely consistent with prior prognostic models. The BODE, ADO, and DOSE indices found the following predictors to be significant: BMI, FEV_1_ (percent predicted), Dyspnoea, Exercise Capacity, Age, Smoking Status, and Exacerbation rate [12–14]. Though age and BMI were rarely selected and we did not have sufficient exercise capacity measurements, FEV_1_, dyspnoea, smoking status, and prior hospitalisations (as a binary instead of a rate) were all consistently included as predictors for both sexes. We used hospitalisations with COPD diagnosis in the past year as a proxy for prior exacerbations and found it to be a strong predictor of future hospitalisations at all timepoints in males and females, supporting existing literature [10].

The strongest additional feature identified in our studies, significant in both sexes and all models, was decreased DLCO_c_ as measured at the first visit to a pulmonary lab after COPD diagnosis. There is growing evidence demonstrating its association with exacerbation rate independently of emphysema [42] and all-cause mortality [43, 44], even in patients with otherwise mild symptoms [45]. The use of haemoglobin-corrected DLCO was chosen to emphasise the effect of pulmonary changes over system effects such as anaemia. DLCO can also be adjusted for body surface area and alveolar volume, though it is unclear if one approach holds a significant advantage over the others.

Survival modelling reveals DLCOc works best as a predictor for adverse events in the short term, as opposed to the other selected predictors which did not violate proportional hazards assumption and therefore can represent chronic risk. Some hypotheses include lower DLCOc patients being more correlated with patients who respond well to treatment or are likely to have consistent lifestyle changes, that most patients with low DLCOc result in quick mortality, but that there is an associated but unobserved effect which can protect within these patients for the long term. DLCOc is known to be a slow changing biomarker, and unlike spirometry which will consistently decline with age, DLCOc is relatively stable within a healthy population. Therefore, sudden or rapid changes in DLCOc can be indicative of a high short-term risk in mortality, and it may be necessary to perform regular DLCOc measurements to manage patient health.

Decreased SpO_2_ was a consistent predictor of hospitalisation and mortality in both sexes. The measurement itself is a non-invasive, surrogate measure for arterial oxygen saturation (SaO_2_), and long-term oxygen therapy is recommended for patients whose SaO_2_ drops below 88% [16]. SpO_2_ has been used as an independent predictor of all-cause hospital mortality [46], and when combined with the BODEX index component increased the ability to predict all-cause mortality [47]. Because of the highly skew nature truncated at 100%, and its clinical use as a binary recommender for oxygen therapy, an argument can be made that SpO_2_ should be discretised for clinical relevance and ease of interpretation especially in comparison to the log(101-SpO_2_) transform employed in this study.

Forced expiratory ratios FEV_1_/FVC and FEV_1_/FEV_6_ were included consistently selected predictors of hospitalisation and mortality in both sexes with no obvious preference of one over the other. The ratios are highly collinear and largely interchangeable for the diagnosis of COPD [48] and it has been established that spirometric measures, including the FEV_1_/FVC ratio, are predictive of future exacerbation [49]. We add to this literature by confirming that FEV_1_/FEV_6_ can aid hospitalisation risk prognosis in men in a similar manner as FEV_1_/FVC but provide no further comments on the choice of one over the other.

Validation and updating of prognostic scores developed in other European ancestry populations [13, 34, 50] within our contemporary Australian cohort is important to assess the effects of ‘dataset drift’ [51] which can lead to degradation in performance when moving a prognostic model outside of its developmental cohort. Differences in population demographics [52] and clinical practices for patient care [53] are primary causes of dataset drift. Our clinical cohort had a higher BMI, lower average mMRC, and lower rate of smoking history than previous prognostic studies. In the same way, our own locally developed model may not generalise well to other populations with different healthcare settings or ethnic makeup, and external validation would be needed.

Our decision to stratify models on sex (instead of including as a covariate) was predicated on the known sex differences in prevalence, expression, diagnosis, comorbidities, and outcomes of COPD patients [27, 28], as well as early exploratory tests demonstrating interactions between sex and DLCOc, mMRC, FEV_1_ and smoking status. An alternative approach would have been to use percent predicted versions of the spirometry variables and add interaction effects to the categorical variables. Future work may benefit from directly comparing the generalisability and equity of sex-specific stratified models against interaction-adjusted models. Further, we acknowledge that use of percent predicted values for spirometry has benefits for cross-study comparisons, and percent predicted values will be pursued in future validation studies.

Neither DOSE nor ADO indices stratified by sex, the DOSE index instead adjusting covariates by age and sex, and ADO concluding that including sex as a covariate did not provide a significant improvement to prognostic performance. Within our cohort, there were differences in prognostic performance between males and females, especially for mortality prediction, but those differences were not significant.

The Cox survival model c-index was in the ballpark of the logistic model AUCs, though c-index and AUC are not directly comparable. The survival model is less at risk of bias from overfitting as it can include all observed mortality events, while also appropriately handling censoring. Competing events from non-COPD-specific mortality were not considered but may be worthwhile to model in the future.

Comorbidities were not given special consideration in general, though we acknowledge that pneumonia in particular was highly prevalent in hospitalisation events with a secondary diagnosis of COPD. The argument to exclude pneumonia cases would help focus prognosis on purely pathophysiological changes, however in practice, distinguishing between pneumonia and acute COPD exacerbations is challenging and could artificially bias the model towards less severe cases.

Long-term prognostic models in general struggle to account for events such as community-acquired pneumonia which cannot be predicted for during baseline. It may be more prudent to instead focus on longitudinal monitoring of patient health and early detection of short-term events. However, long-term prognostic models still have a role to play for patient education and clinical resource allocation, particularly in the context of clinical and drug trials, which benefit greatly from more discriminative models being able to select out a subgroup of patients at highest risk of an event.

We provide, as an example, a potential use case for our binary regression models as a selection criterion for including patients in clinical trials. Say we want to select 200 male and 200 female to test the efficacy of a drug in preventing severe acute exacerbations of COPD, with patients being monitored over the span of one year. Randomly selecting patients with no prior information would result in approximately 20 (10%) and 19 (9.5%) of the selected male and female patients going on to have a COPD-specific hospitalisation. Using the top 200 male and female patients, after ranking by 1-year hospitalisation risk would instead result in 67 (33.5%) and 63 (31.5%) selected male and female patients being hospitalised in 1 year, substantially increasing the power of the experiment.

We intentionally chose to look at patients at or just after first diagnosis of COPD as it is at this time point that prognostic models have the greatest chance to impact clinical decision making. Because COPD is irreversible, most clinical strategies are targeted at the management of symptoms and prevention of exacerbations [9]. A prognostic risk model for future hospitalisation risk could be used to consider recommending stronger pharmacotherapy plans to a patient before repeated exacerbation events have occurred and/or adding other evidence-based therapeutic interventions (e.g., smoking cessation, pulmonary rehabilitation). Additionally, among the patients who continue to smoke after COPD diagnosis [54], clinical engagement with patients has demonstrated improved cessation rates [55–58], and prognostic models could be used to prioritise patients at greatest risk when resources and time are limited. In spite of looking at first diagnosis, approximately one tenth of the cohort had already been hospitalised due to their COPD symptoms suggesting that more efforts are needed to detect patients while they are in the earlier stages of COPD.

There were some limitations to our study. The use of routinely collected retrospective data is subject to variability of collection and reporting standards, although these did not obviously change over the duration of our study. Though we employed state-wide data linkage to acquire hospitalisation and mortality event data for our COPD cohort, these records only cover the public sector within South Australia and the Northern Territory, so it is possible that some hospitalisation events were missed. Small outcome counts in COPD-specific mortality, particularly after 3 years, was a key limitation for model stability and generalisability. The high AUCs reported may not be reproducible on external datasets. The use of ICD-10 codes J44.0-J44.9 to identify COPD-specific hospitalisation and mortality may not properly represent coding practices for COPD and not including codes for emphysema, chronic bronchitis, or asthma may have underrepresented the true exacerbation rate in the cohort. Though, this is mitigated somewhat due to our reliance on spirometry for cohort inclusion. Without external validation, the clinical utility of this study is limited to a south Australian cohort and may not generalise to other healthcare setting, populations, or ethnic groups. Further, the superiority of our local models and the extended ADO and DOSE models may not translate to other cohorts; only external validation will be able to demonstrate this. The temporally distinct hold-out test set was chosen to mitigate this risk, as we are confident that, at least within our own cohort, that our model will be useful for future internal use. Additionally, while great care was taken to collect and clean smoking status, degree of dyspnoea, and SpO_2_ from the clinical datasets, all measurements were contained in free-text data instead of formalised data tables, and inconsistent measurement, reporting, or interpretation may have introduced noise, particularly for smoking status which had many coding variations. While smoking status was accounted for, our dataset did not account for pack years, time since smoking cessation, drug use, or medication, all of which may have contributed to outcome risk.

## Conclusion

Our study describes the development and internal validation of local prognostic models for hospitalisation, and mortality among COPD patients, as well as validation of the updated ADO and DOSE models within a large Australian cohort. Our locally developed models achieved better discrimination, though not always significant, than the existing updated ADO and DOSE indices on a temporally separate internal test set. We identified DLCOc as a strong predictor of COPD-specific hospitalisation and mortality events within a 5-year time period and found ADO and DOSE model fit to improve significantly when extended with DLCOc. External validation is needed to assess whether DLCOc is a consistent predictor in other cohorts and we recommend it be considered for inclusion in future modelling work and monitored more consistently within clinical practice.

## Supplementary Information

Supplementary Material 1.

## Data Availability

The data that support the findings of this study are available from the Southern Adelaide Local Health Network (SALHN), but restrictions apply to the availability of these data, which were used under license for the current study, and so are not publicly available. Data are however available from the authors upon reasonable request and with permission of SALHN.

## Abbreviations

ADO: Age, Dyspnoea, and airflow Obstruction
AUC, AUROC: Area Under (Receiver Operator) Characteristic Curve
BMI: Body Mass Index
BODE: Body-mass index, airflow Obstruction, Dyspnoea, and Exercise capacity
BODEX: Body-mass index, airflow Obstruction, Dyspnoea, Exacerbations
CIL: Calibration In the Large
COPD: Chronic Obstructive Pulmonary Disease
DALY: Disability Adjusted Life Years
DLCOc: Diffusing capacity of Lung for Carbon Monoxide (CO) corrected for haemoglobin
DOSE: Dyspnoea, airflow Obstruction, Smoking Status, and Exacerbation frequency
EPV: Events Per Variable
FEV_1_, FEV_6_: Force Expiratory Volume in 1 and 6 s
FIVC: Forced Inspiratory Vital Capacity
FVC: Force Vital Capacity
ICD-10: International Statistical Classification Disease, 10th Edition
IQR: Interquartile Range
MICE: Multivariate Imputation by Chained Equations
MMEF: Maximal Mid-Expiratory Flow
mMRC: Modified Medical Research Council
PEF: Peak Expiratory Flow
ROC: Receiver Operator Characteristic
SaENET: Stacking adaptive Elastic Net
SAC HREC: Southern Adelaide Clinical Human Research Ethics Committee
SALHN: Southern Adelaide Local Health Network
SaO_2_: Saturation of Arterial Oxygen
SpO_2_: Saturation of Peripheral Oxygen
