# Supplementary material for: Prognostic models for mortality and hospitalisation risk in a contemporary Australian chronic obstructive pulmonary disease cohort
Source: Respir Res. 2026 Feb 5;27:118. doi: 10.1186/s12931-026-03531-7 (PMC12964674; doi:10.1186/s12931-026-03531-7)
Supplement: Supplementary file 1 — Supplementary Material 1. [file 12931_2026_3531_MOESM1_ESM.docx]

# Missing Values

**Table S1: Percent missingness for each variable from dataset.**

| **Variable** | **Percentage missing (%)** |
| --- | --- |
| **Age** | 0.00 |
| **Height** | 0.00 |
| **Weight** | 0.00 |
| **BMI** | 0.00 |
| **FEV_1_** | 0.00 |
| **FVC** | 0.00 |
| **FIVC** | 31.29 |
| **FEV_1_/FVC** | 0.00 |
| **FEV_1_/FEV_6_** | 0.30 |
| **PEF** | 0.00 |
| **MMEF** | 0.07 |
| **DLCOc** | 13.88 |
| **SpO_2_** | 26.04 |
| **Hospitalisation (Past Year)** | 0.00 |
| **mMRC** | 12.46 |
| **Smoking Status** | 6.08 |

DLCOc=Diffusing capacity of Lungs for Carbon Monoxide corrected for haemoglobin; FEV_1_=Forced Expiratory Volume in 1 second; FVC=Forced Vital Capacity; MMEF=Maximal Mid-Expiratory Flow; mMRC=modified Medical Research Council Questionnaire Score.

**Table S2 Number of outcome events and patients in the training and testing splits for each outcome split by sex.**

|  | Number of Events / Number of Patients (%) | |
| --- | --- | --- |
| Train/Test – Outcome | **Female** | **Male** |
| Train - Hosp. 1 year | 470 / 2,588 (18.2%) | 503 / 3,434 (14.6%) |
| Test – Hosp. 1 year | 107 / 1,095 (9.8%) | 141 / 1,356 (10.4%) |
| Train – Hosp. 3 year | 674 / 2,502 (26.9%) | 757 / 3,317 (22.8%) |
| Test – Hosp. 3 year | 223 / 1,092 (20.4%) | 283 / 1,354 (20.9%) |
| Train – Hosp. 5 year | 647 / 2,006 (32.3%) | 760 / 2,728 (27.9%) |
| Test – Hosp. 5 year | 351 / 1,198 (29.3%) | 386 / 1,443 (26.7%) |
| Train – Mort. 3 year | 96 / 2,502 (3.8%) | 120 / 3,317 (3.6%) |
| Test – Mort. 3 year | 26 / 1,092 (2.4%) | 50 / 1,354 (3.7%) |
| Train – Mort. 5 year | 135 / 2,006 (6.7%) | 185 / 2,728 (6.8%) |
| Test – Mort. 5 year | 60 / 1,198 (5.0%) | 83 / 1,443 (5.8%) |
| Train – Survival | 343 / 2,953 (11.6%) | 454 / 3,909 (11.6%) |
| Test - Survival | 97 / 774 (12.5%) | 101 / 942 (10.7%) |

**Table S3. Coefficients for extended DOSE and updated ADO models**

|  | Intercept | Score | DLCOc | D2.pvalue |
| --- | --- | --- | --- | --- |
| DOSE |  |  |  |  |
| Hosp. 1 year | -2.35 | 0.309 | -1.32 | <0.001 |
| Hosp. 3 years | -1.77 | 0.286 | -1.61 | <0.001 |
| Hosp. 5 years | -1.45 | 0.293 | -1.39 | <0.001 |
| Mort. 3 years | -5.11 | 0.337 | -3.42 | <0.001 |
| Mort. 5 years | -4.20 | 0.267 | -3.42 | <0.001 |
| Updated ADO |  |  |  |  |
| Hosp. 1 year | -3.02 | 0.333 | -1.24 | <0.001 |
| Hosp. 3 years | -2.52 | 0.339 | -1.46 | <0.001 |
| Hosp. 5 years | -2.08 | 0.309 | -1.26 | <0.001 |
| Mort. 3 years | -6.67 | 0.577 | -3.18 | <0.001 |
| Mort. 5 years | -5.37 | 0.440 | -3.21 | <0.001 |

# Imputations

| 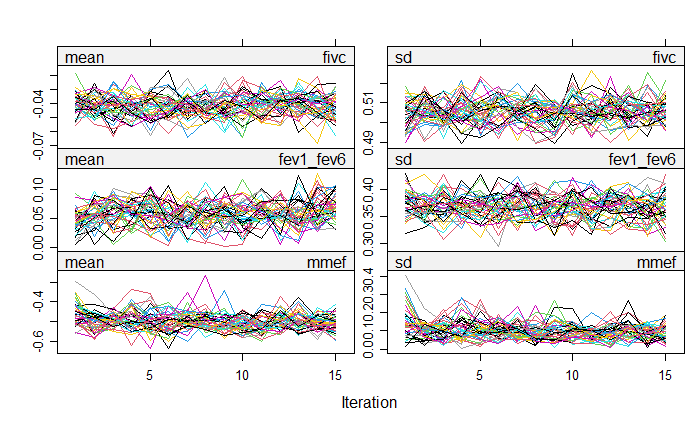 | 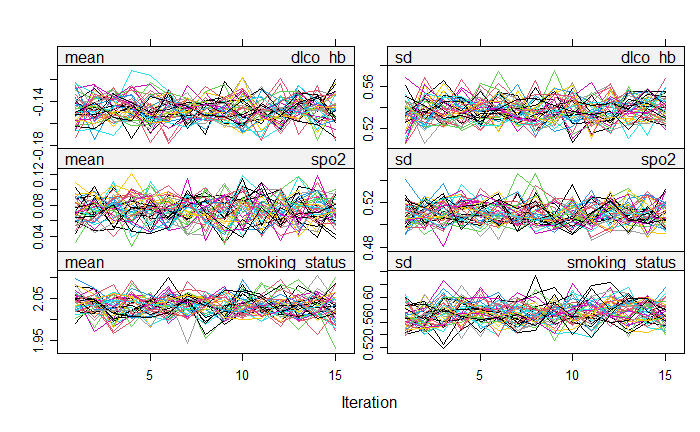 |
| --- | --- |
| 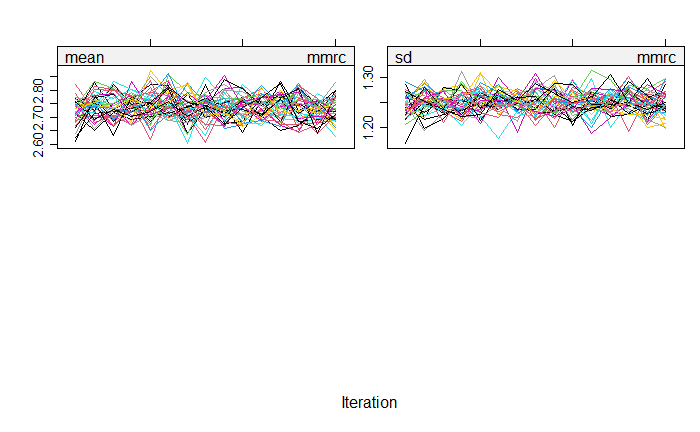 |  |

**Figure S1: Imputation chains generated by MICE for m=10 imputations.**

**Figure S2: Densities of imputed variables (red) vs observed variables (blue).**

**
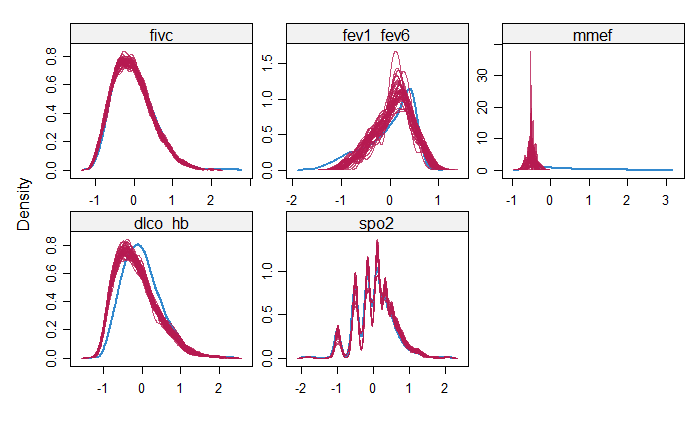
**

Some observations: Imputed MMEF distribution does not match the observed distribution because so few imputations are required. Similarly, FEV_1_/FEV_6_ has a noisy distribution because few imputations were needed. noisier on their imputed densities due to needing very few imputed rows, and so those imputed values are not representative of the sample distributions they came from. SpO_2_, because of the log transform on discrete data (values recorded to integer precision), has large gaps between lower values but smooths at the right end where the log differences are smaller and better approximate a continuous distribution. The variables with the largest amount of missingness, FIVC and DLCOc (encoded as dlco_hb in the dataset) have imputed distributions very closely matching their observed distributions.

# Logistic models for hospitalisation predictions

**Figure S3: Cross validated performances from penalised logistic models for hospitalisation after 1 year.**

| **Female** | **Male** |
| --- | --- |
| 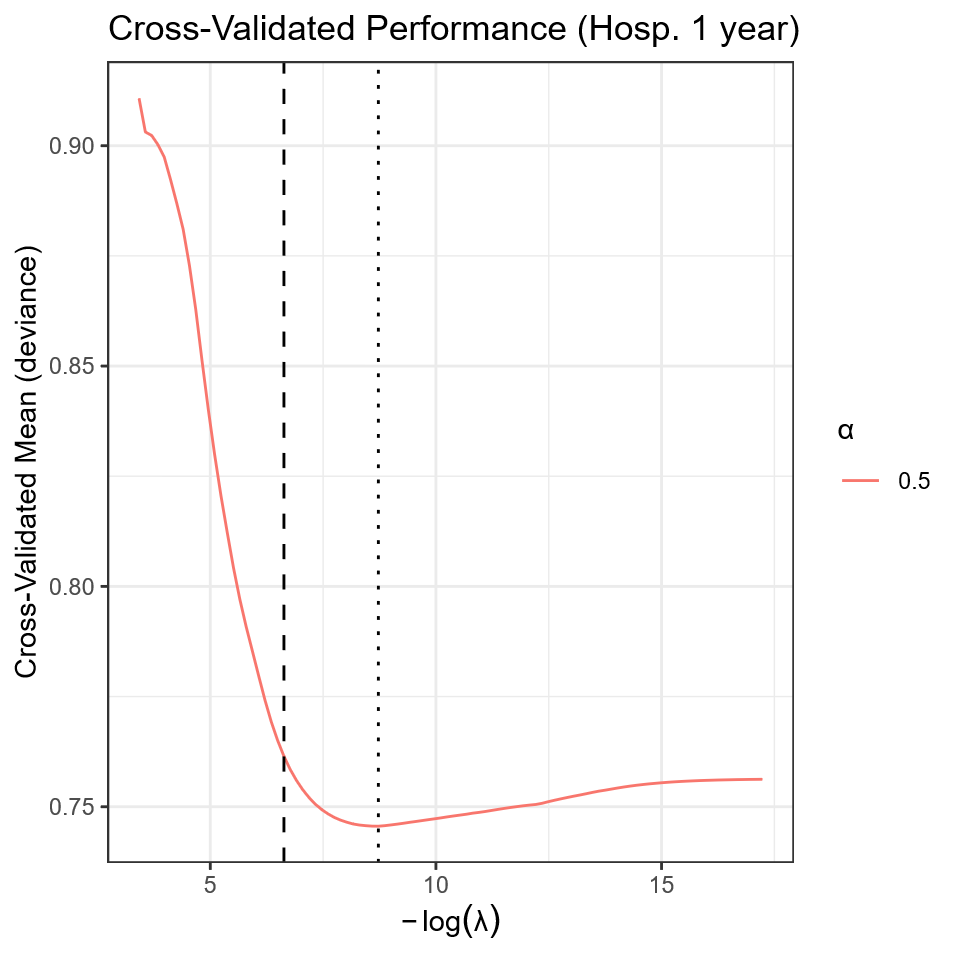 | 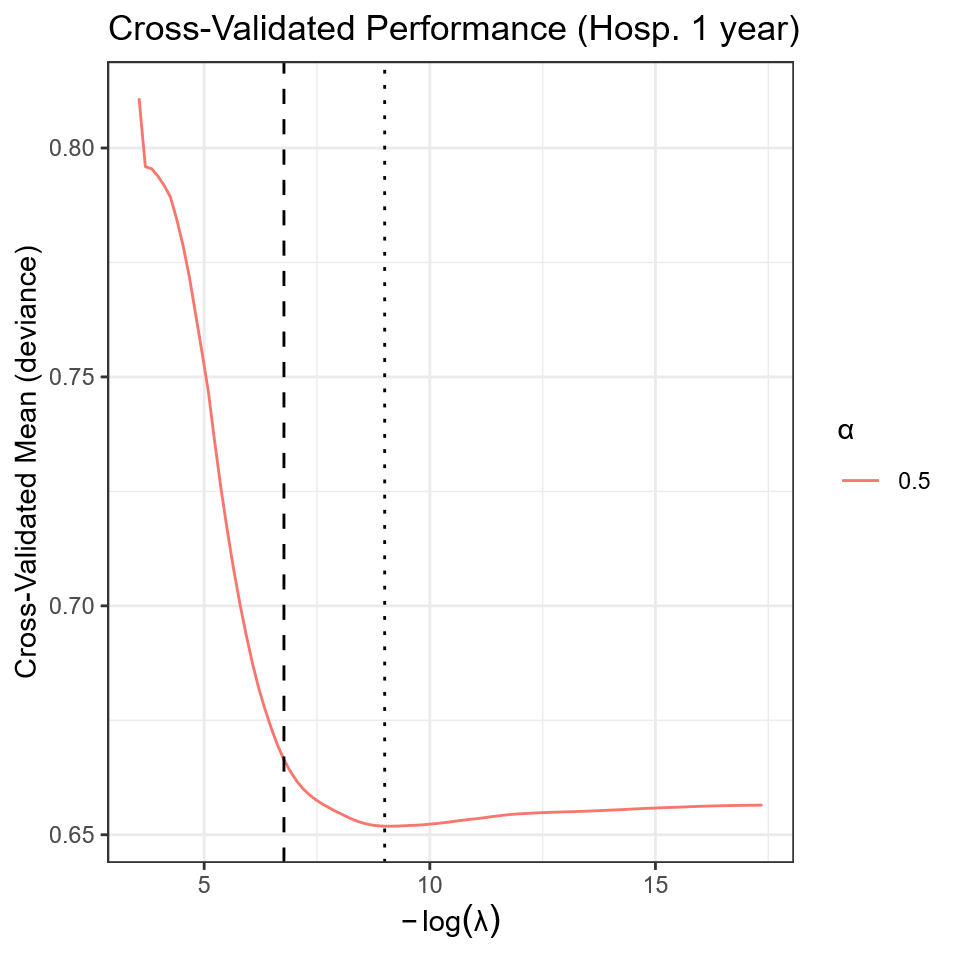 |

**Figure S4: Coefficient paths from penalised logistic models for hospitalisation after 1 year.**

| **Female** | **Male** |
| --- | --- |
| 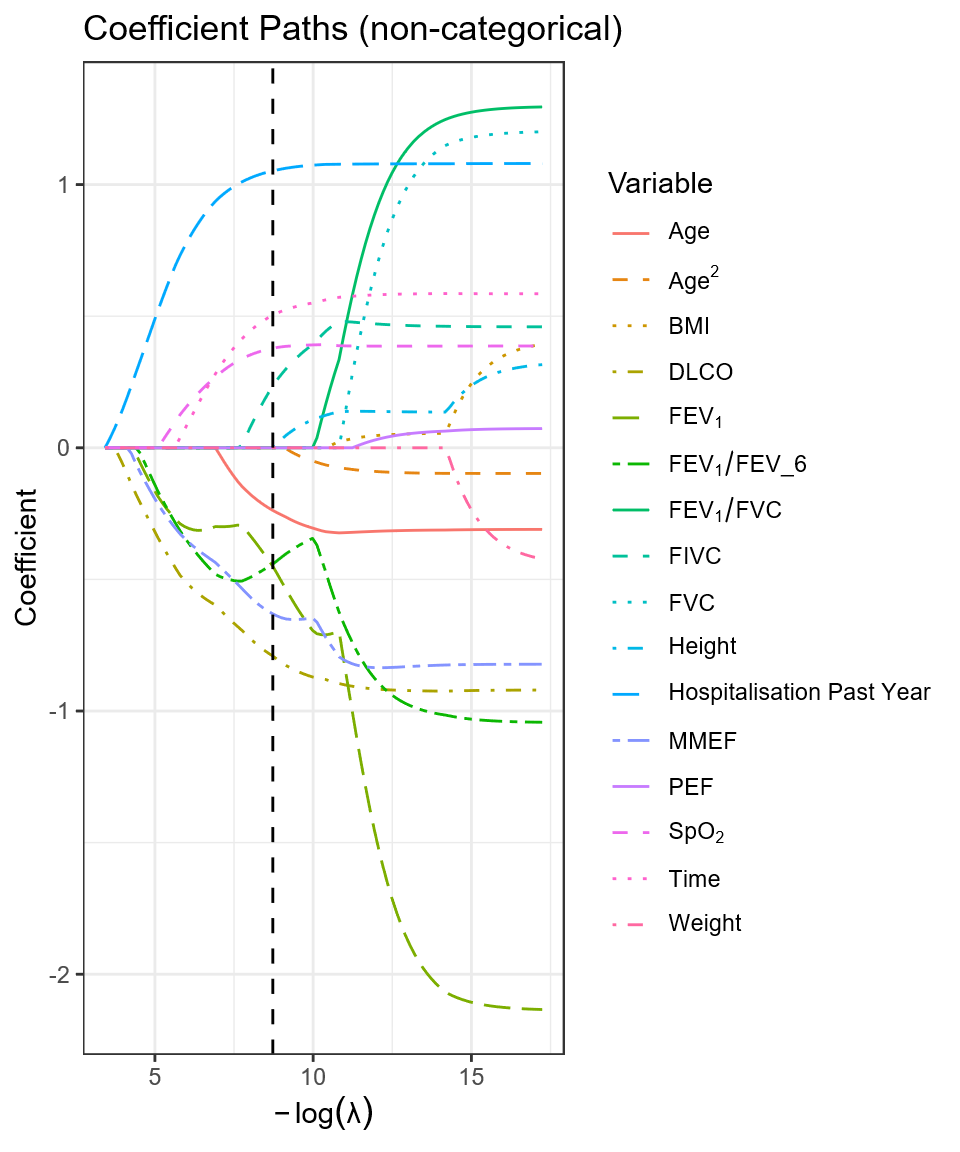 | 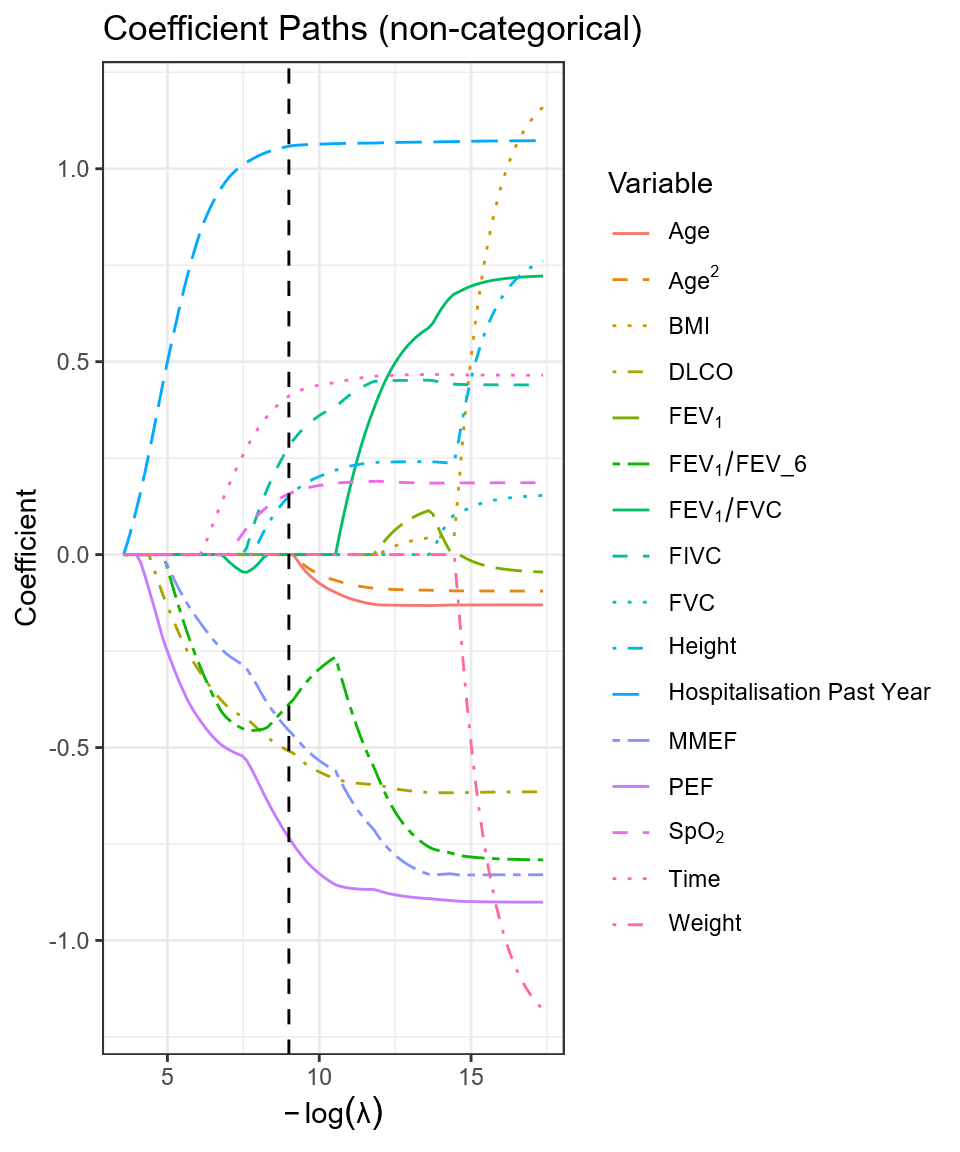 |
| 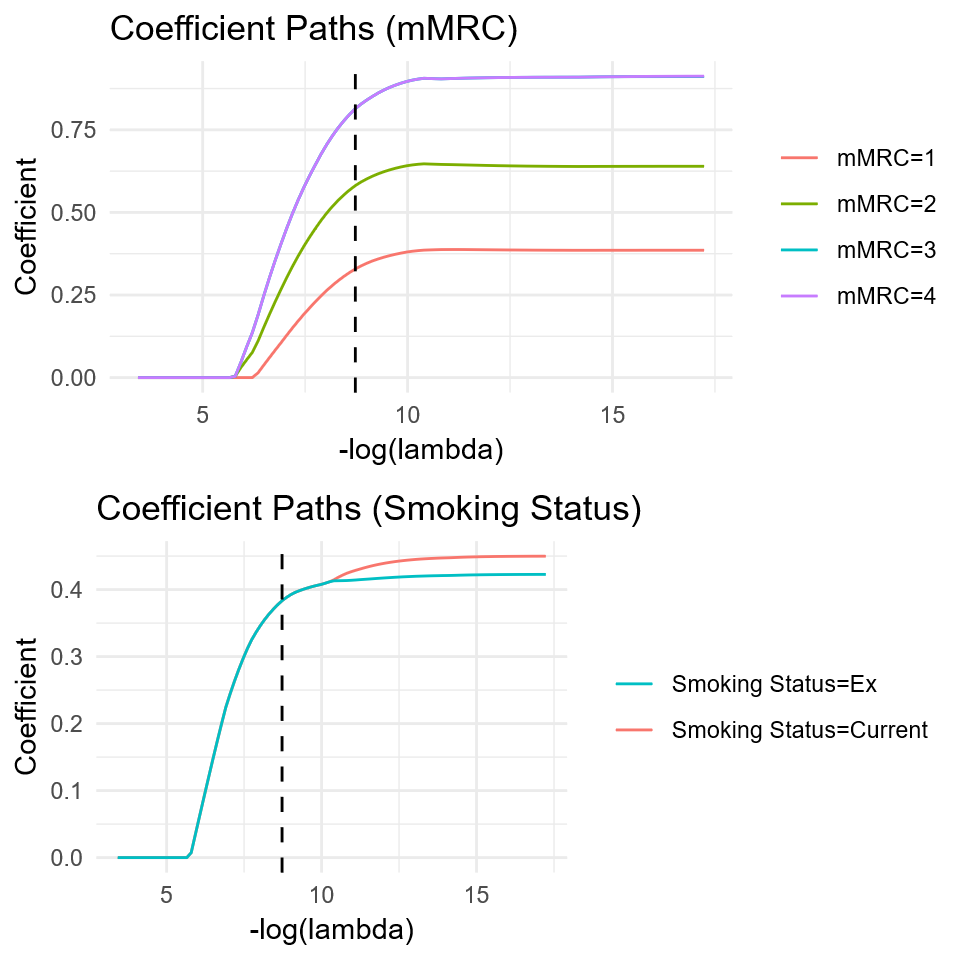 | 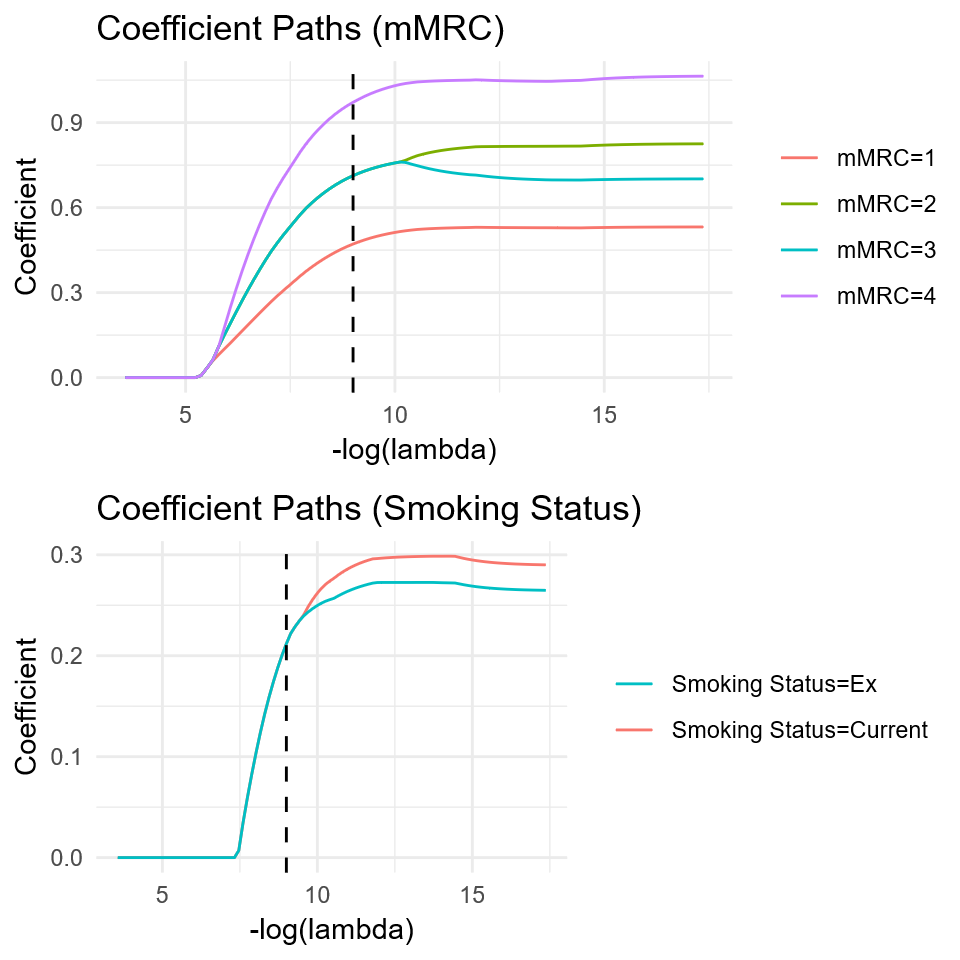 |

**Figure S5: Cross validated performances from penalised logistic models for hospitalisation after 3 years.**

| **Female** | **Male** |
| --- | --- |
| **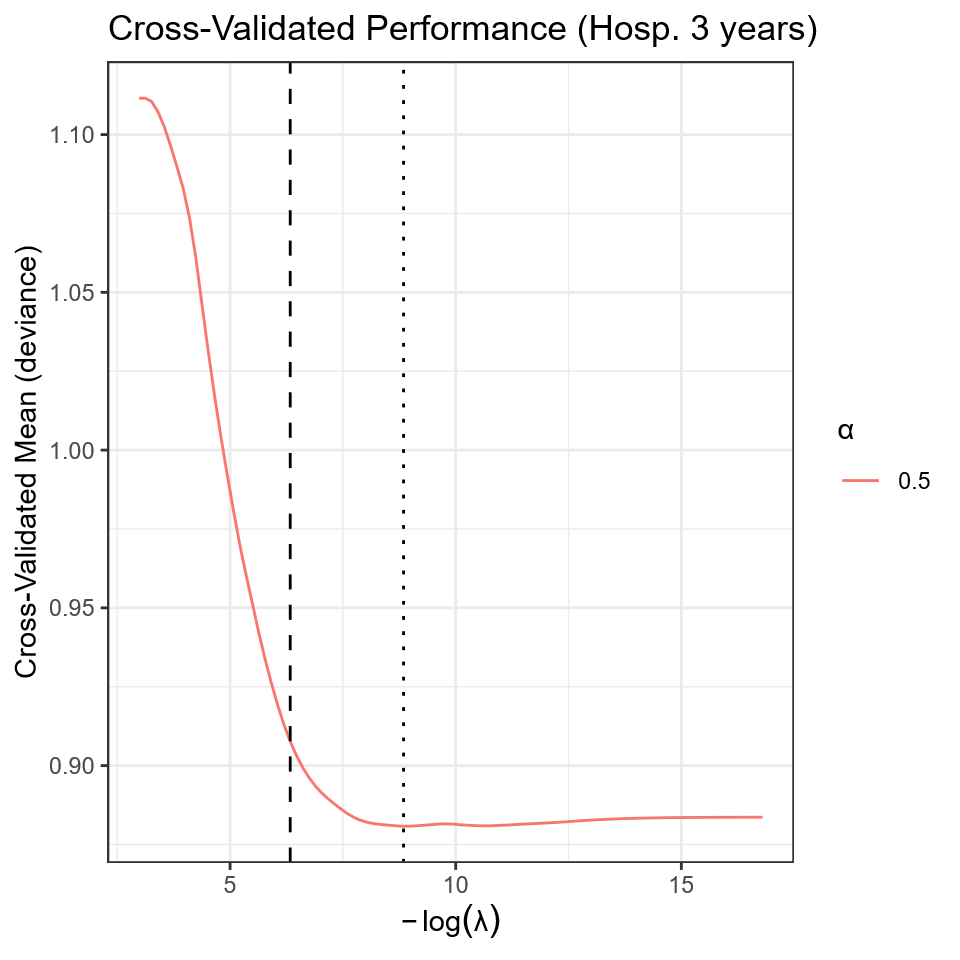** | 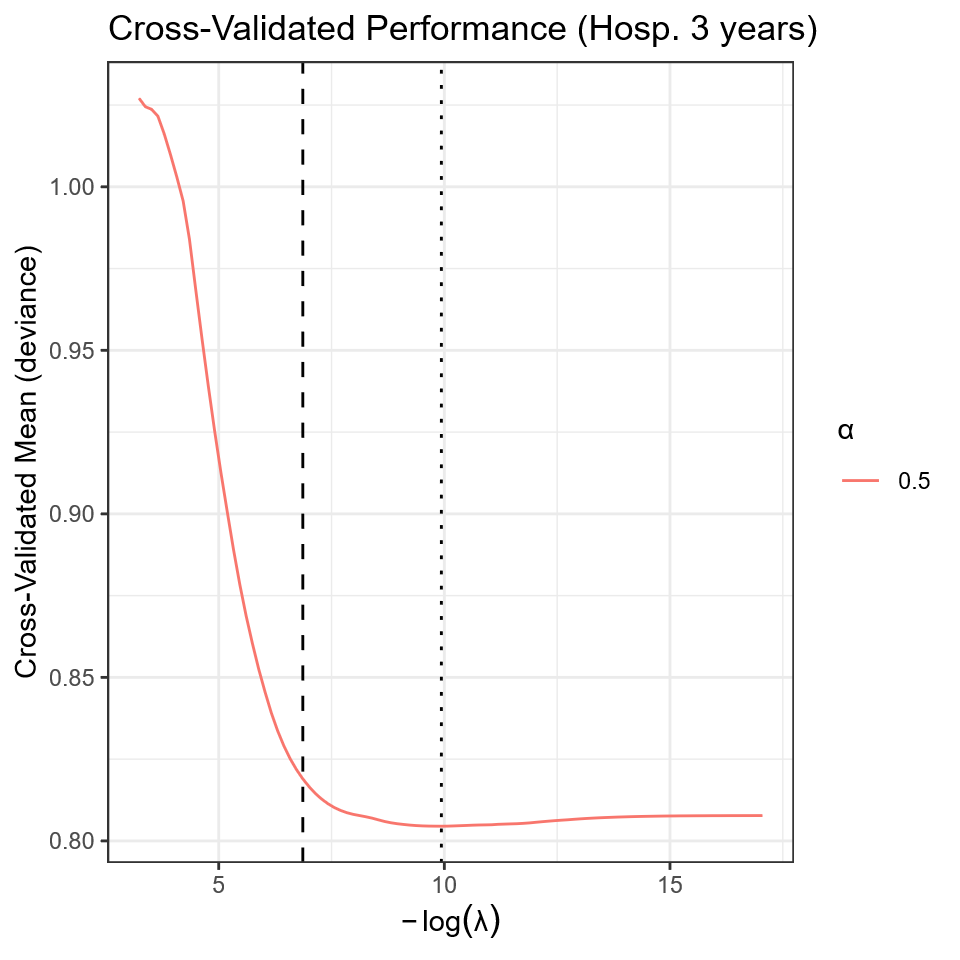 |

**Figure S6: Coefficient paths from penalised logistic models for hospitalisation after 3 years.**

| **Female** | **Male** |
| --- | --- |
| 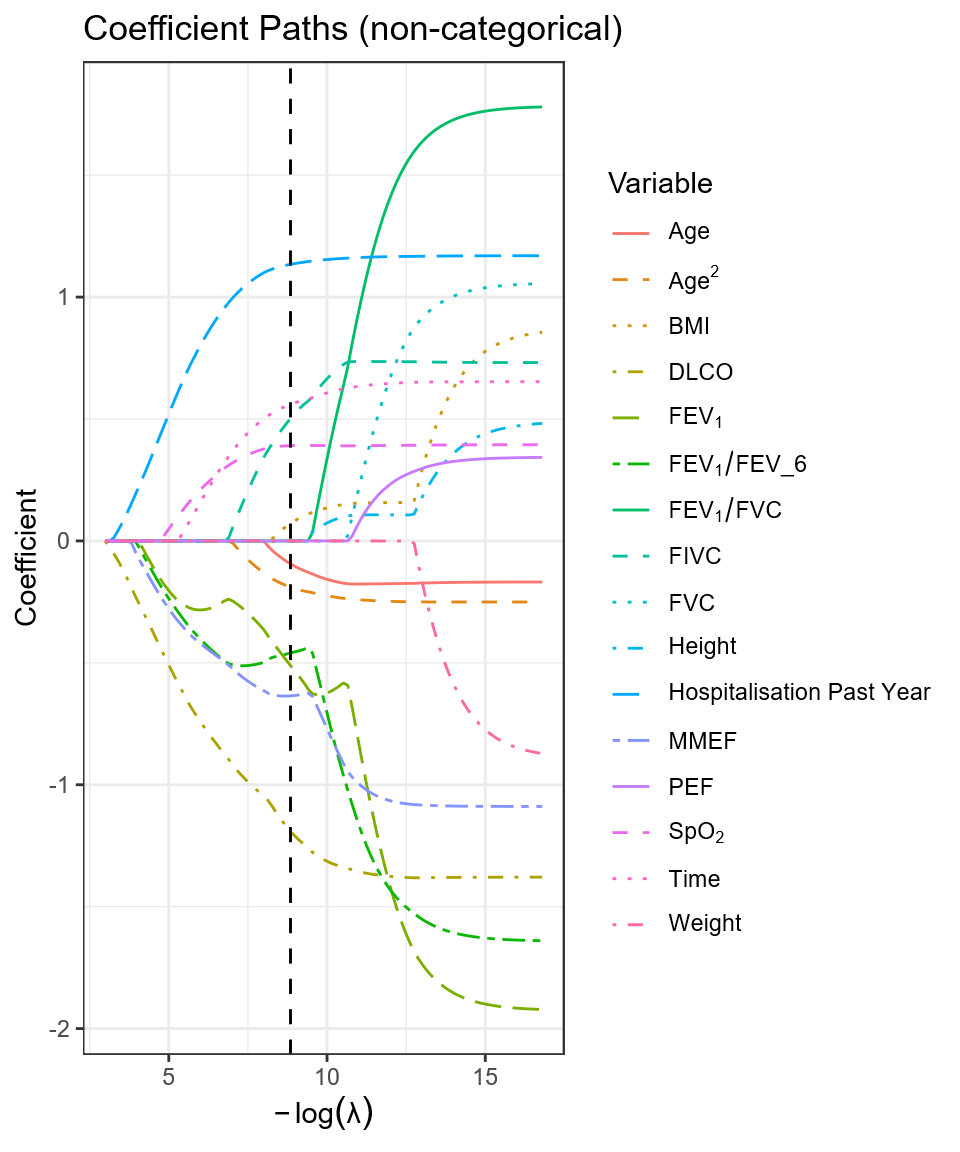 | 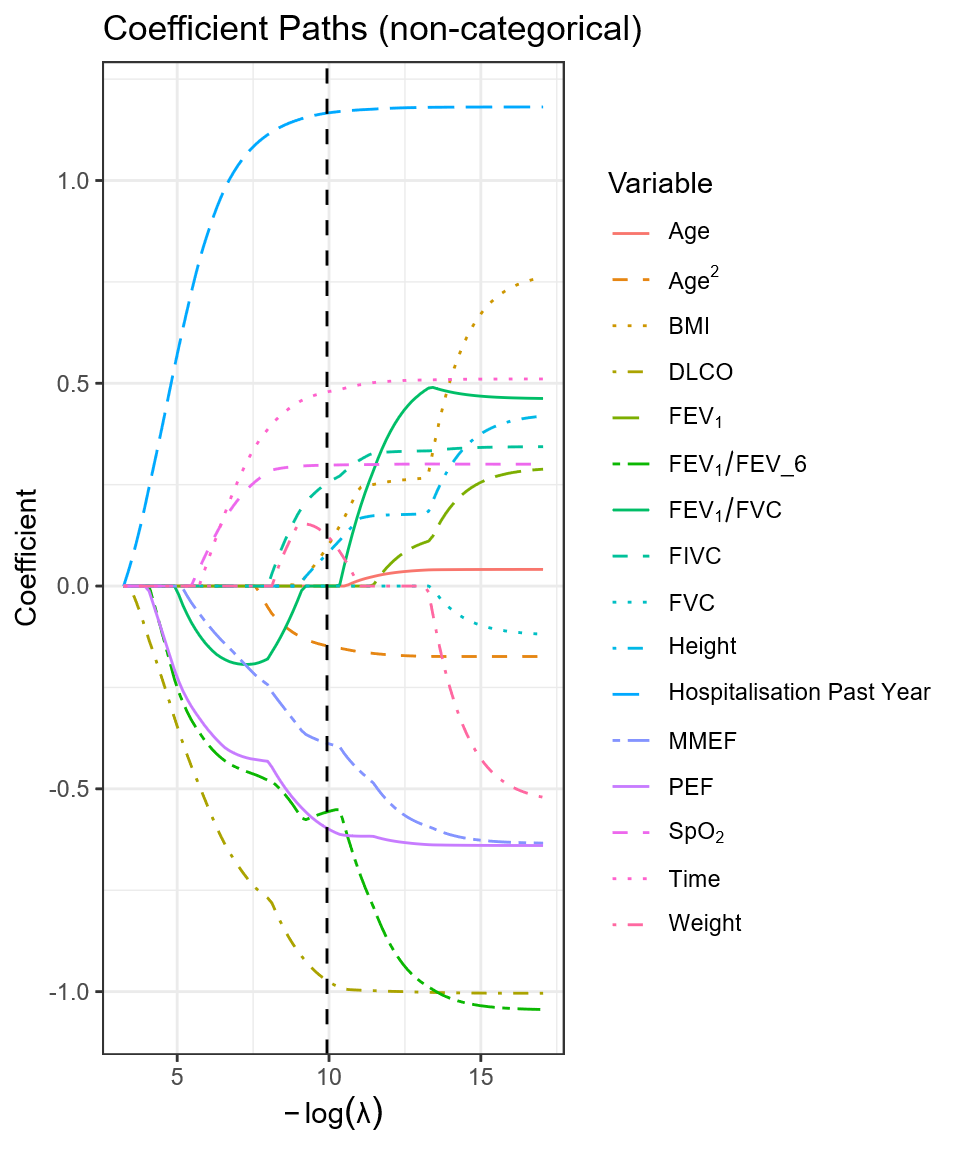 |
| 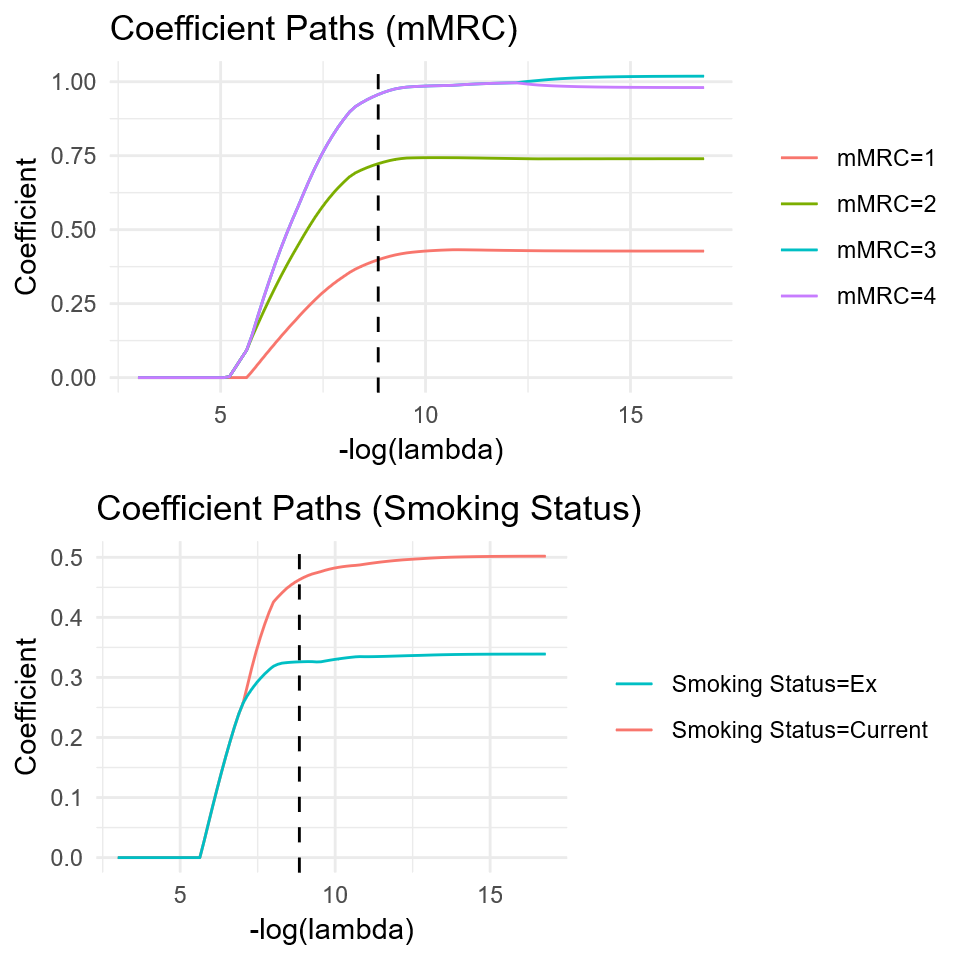 | 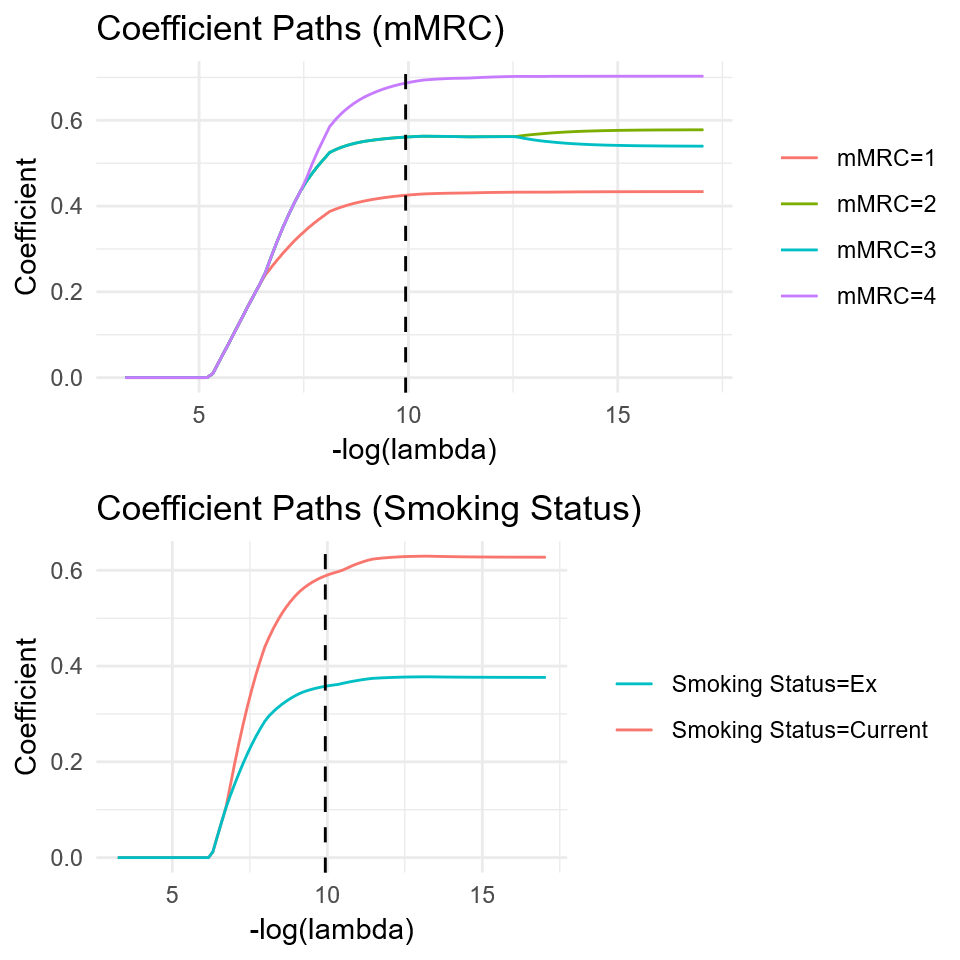 |

**Figure S7: Cross validated performances from penalised logistic models for hospitalisation after 5 years.**

| **Female** | **Male** |
| --- | --- |
| 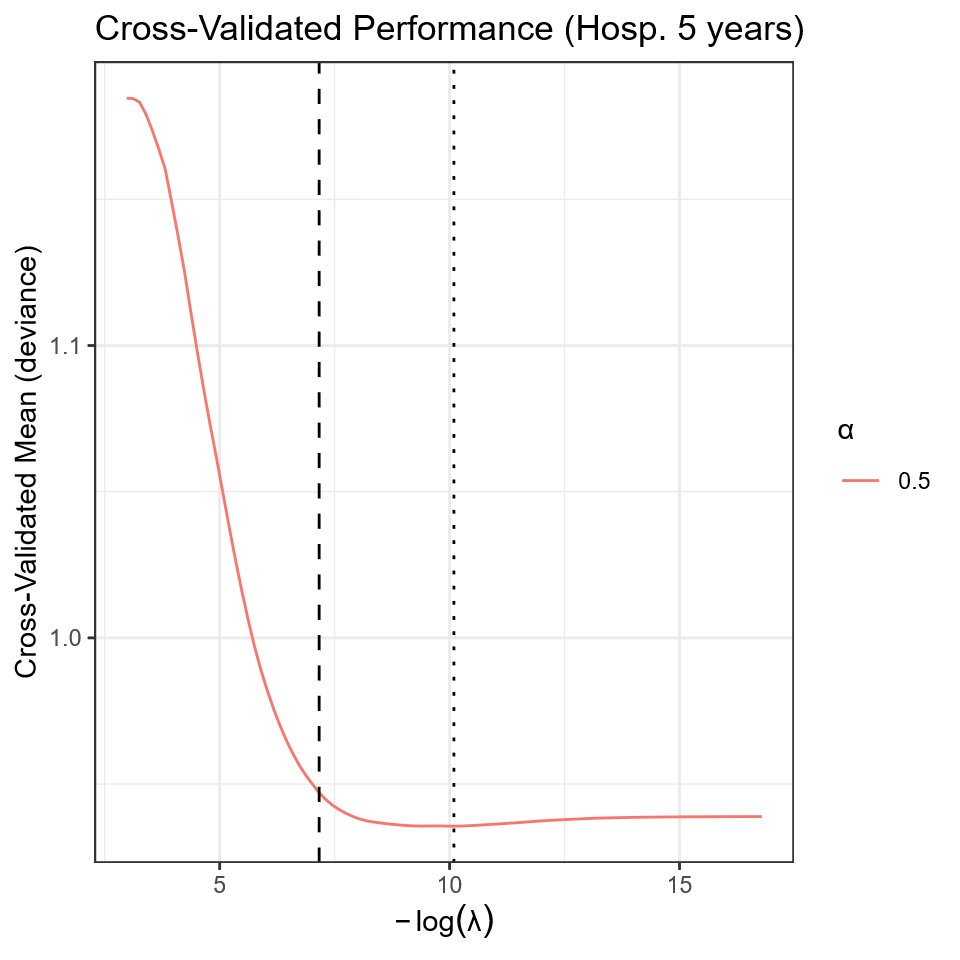 | 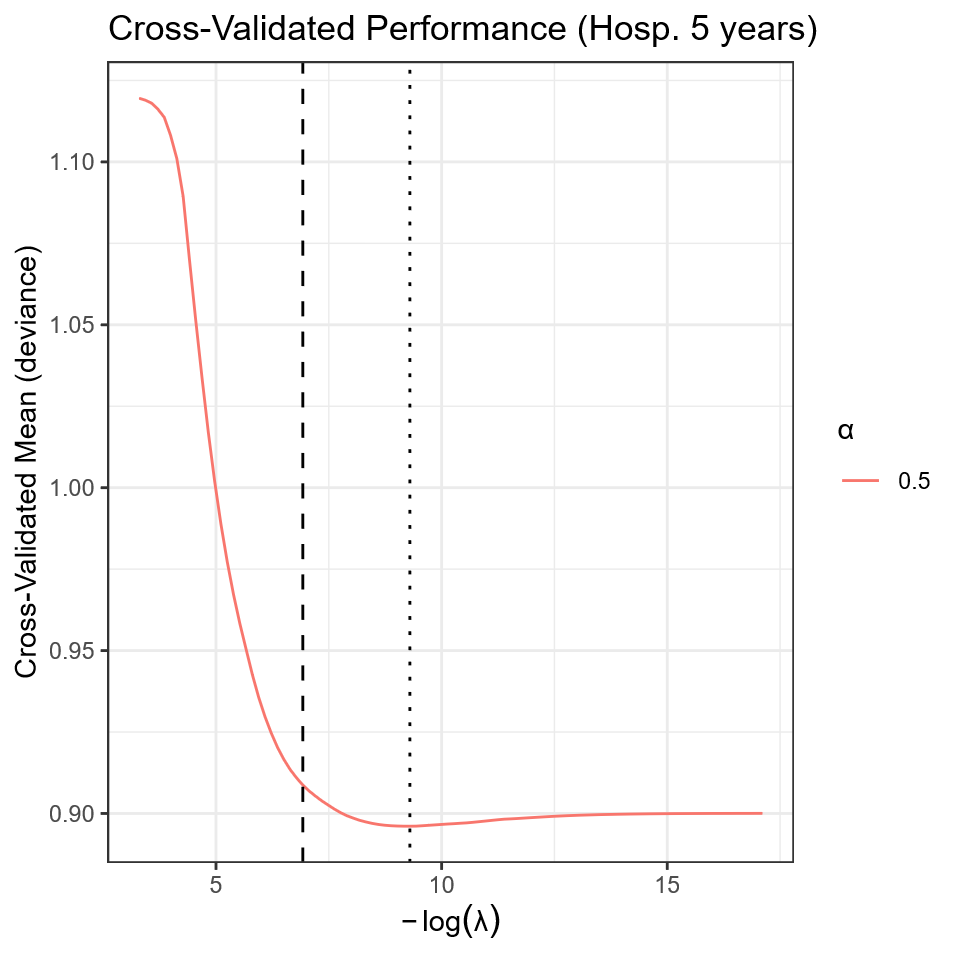 |

**Figure S8: Coefficient paths from penalised logistic models for hospitalisation after 5 years**

| **Female** | **Male** |
| --- | --- |
| 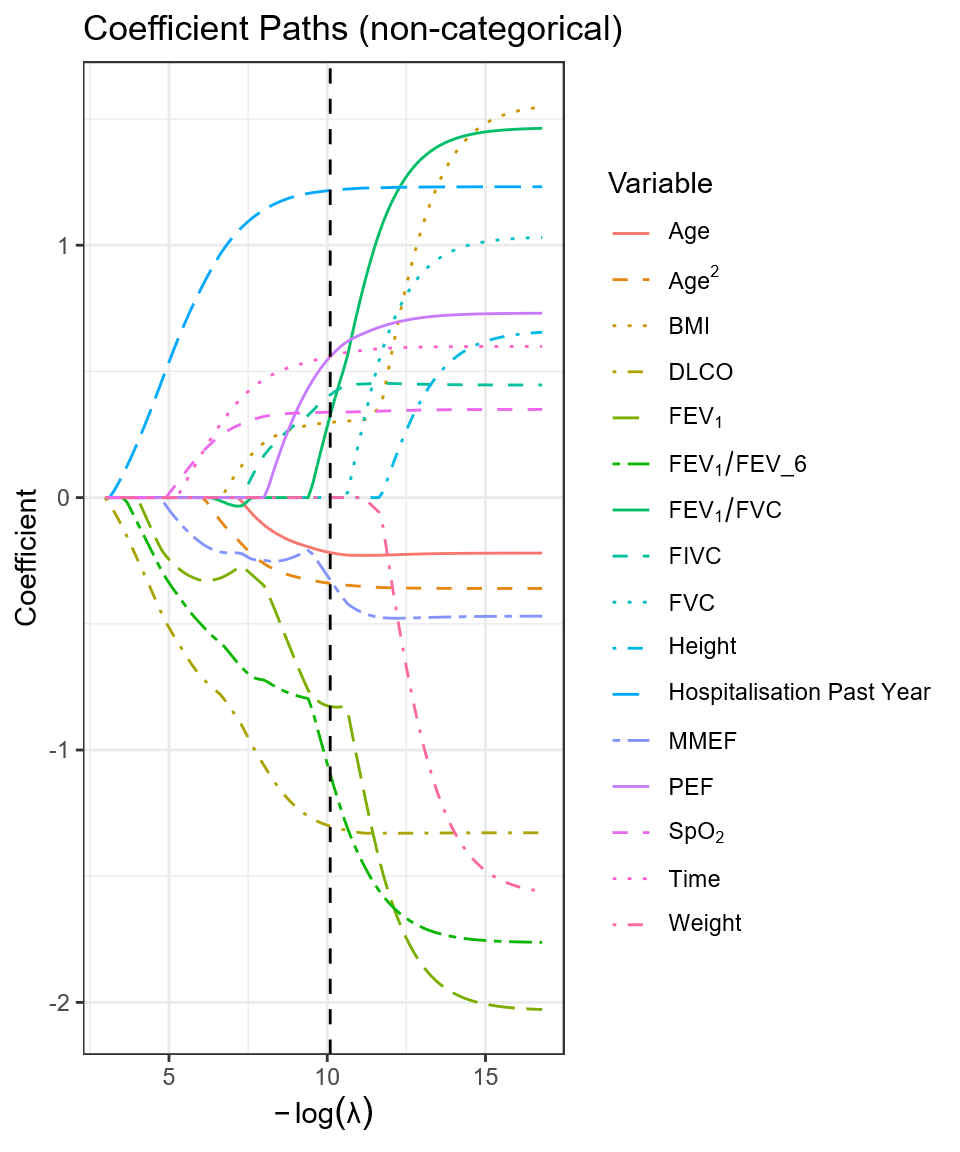 | 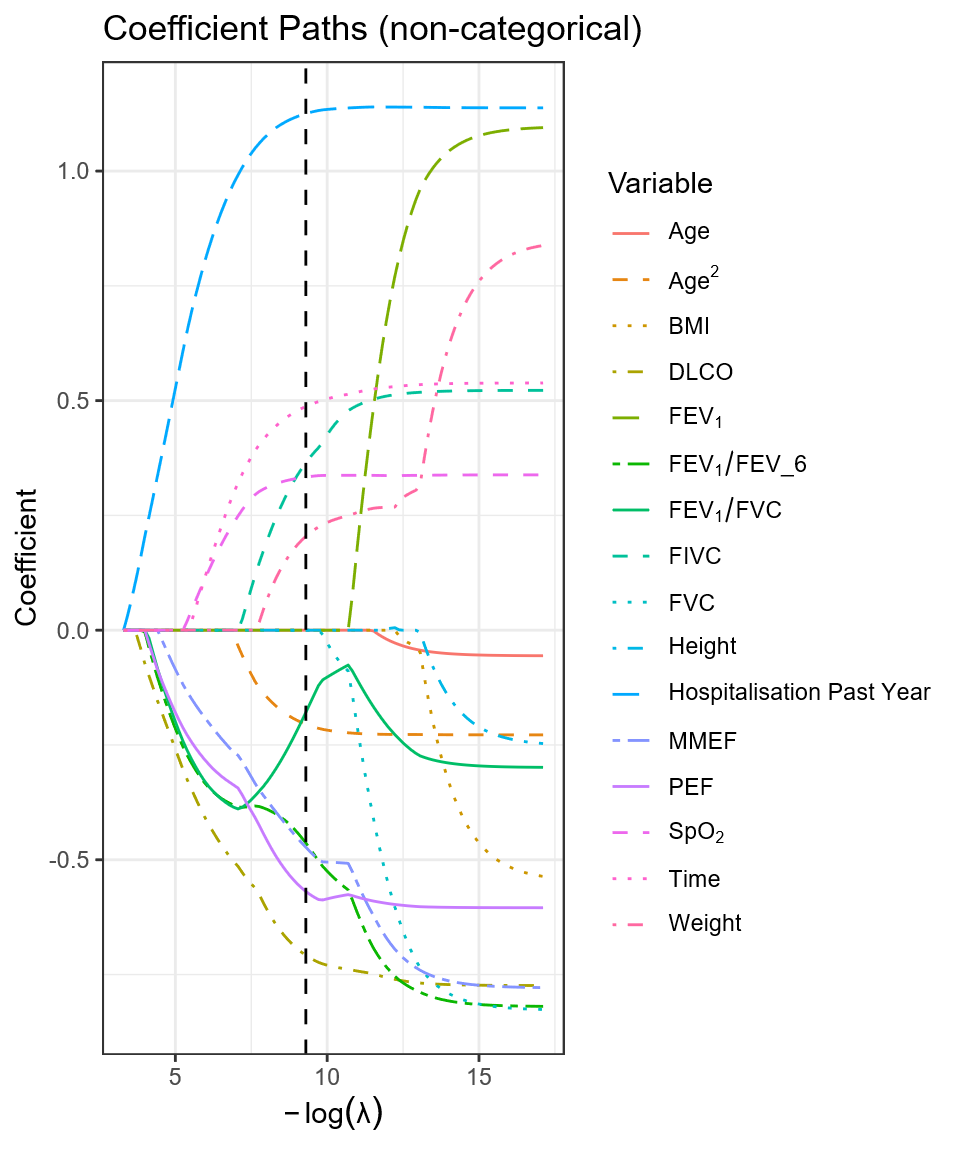 |
| 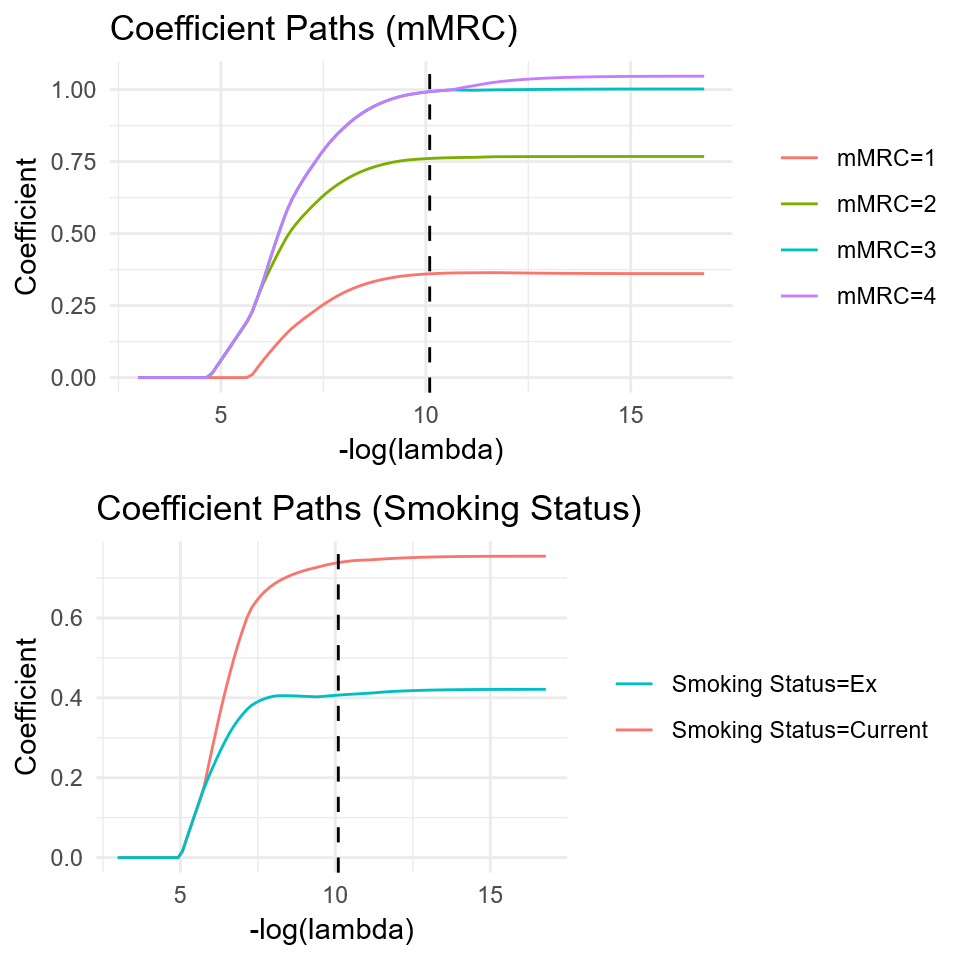 | 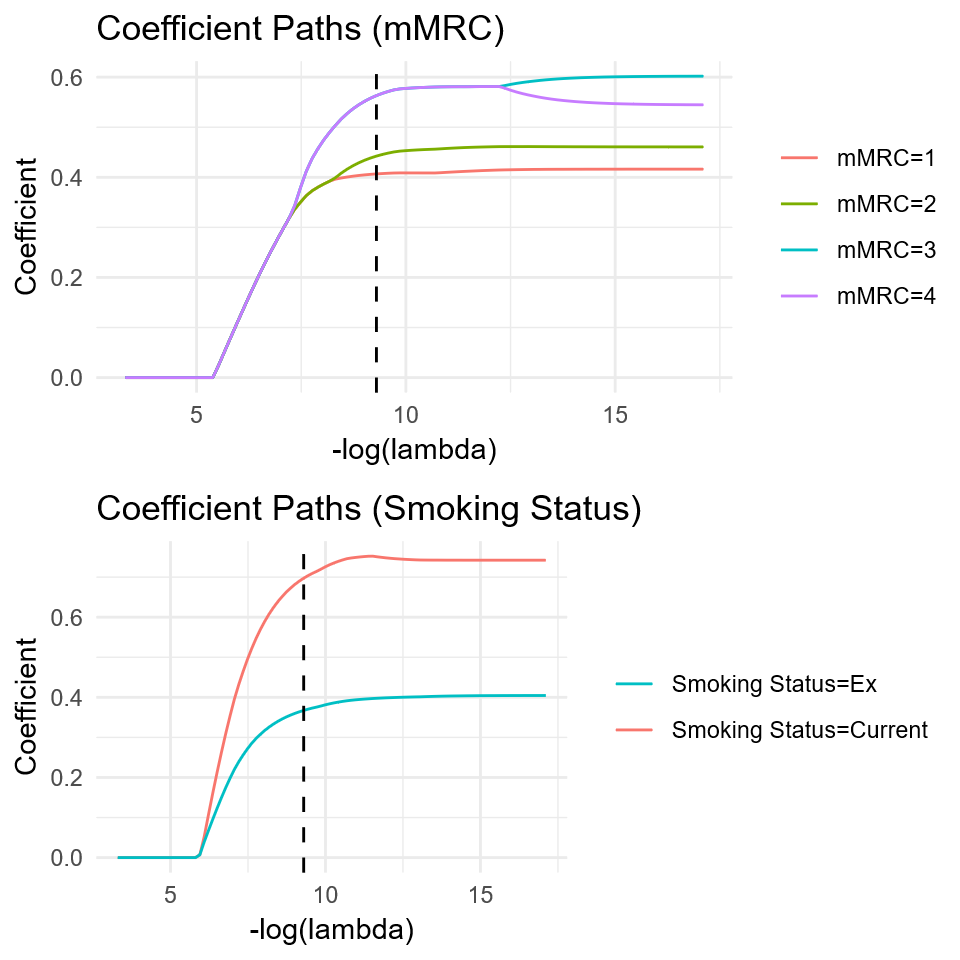 |

# Logistic models for mortality predictions

**Figure S9: Cross validated performances from penalised logistic models for mortality after 3 years.**

| **Female** | **Male** |
| --- | --- |
| 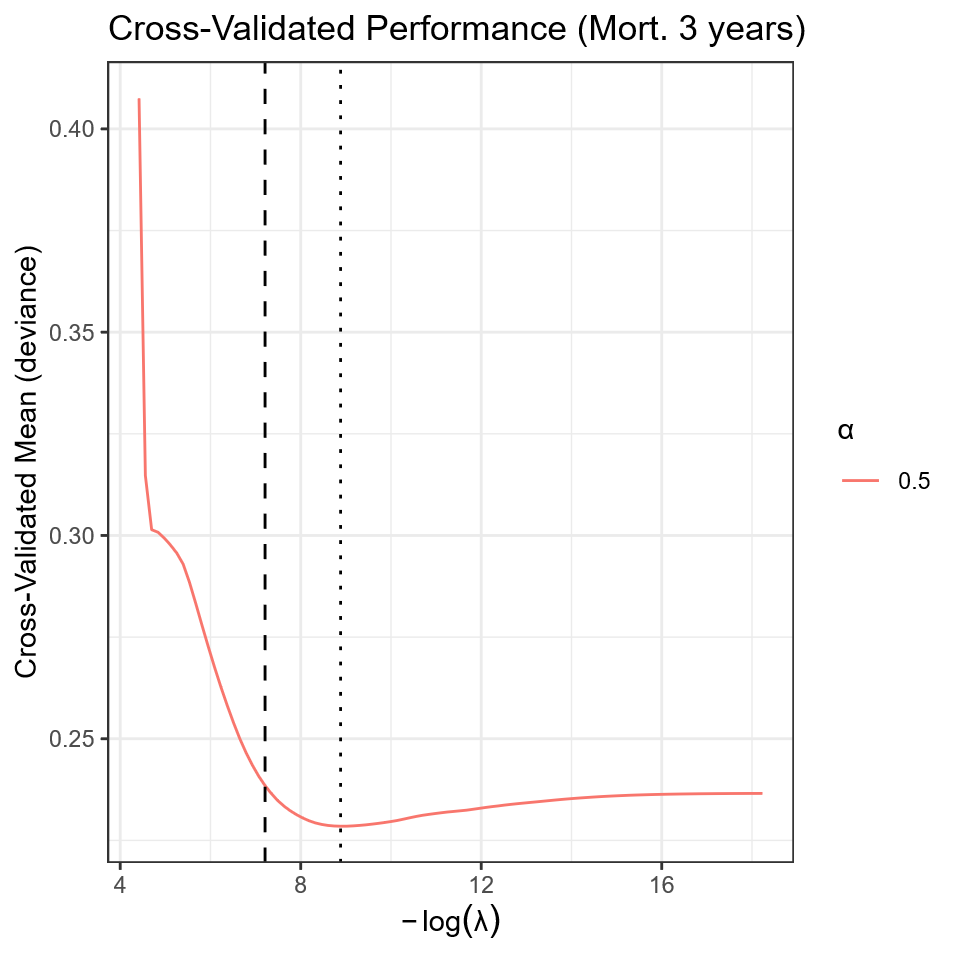 | 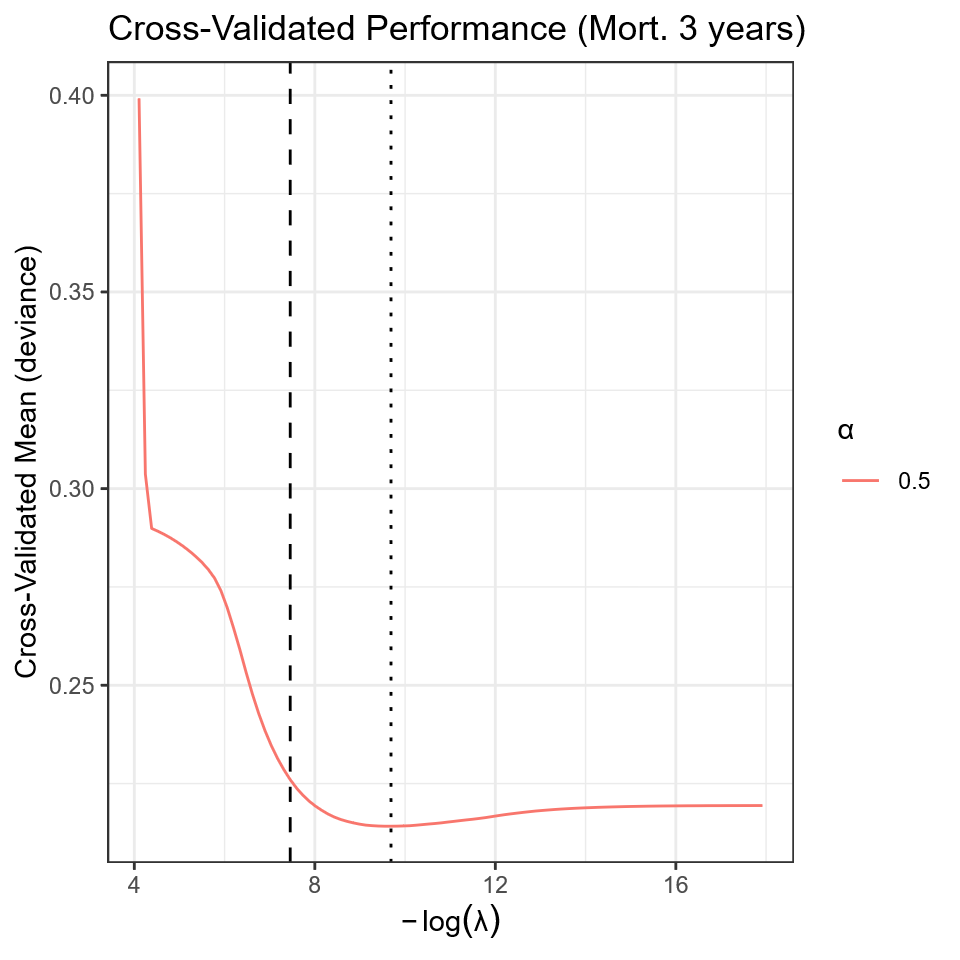 |

**Figure S10: Coefficient paths from penalised logistic models for mortality after 3 years.**

| **Female** | **Male** |
| --- | --- |
| 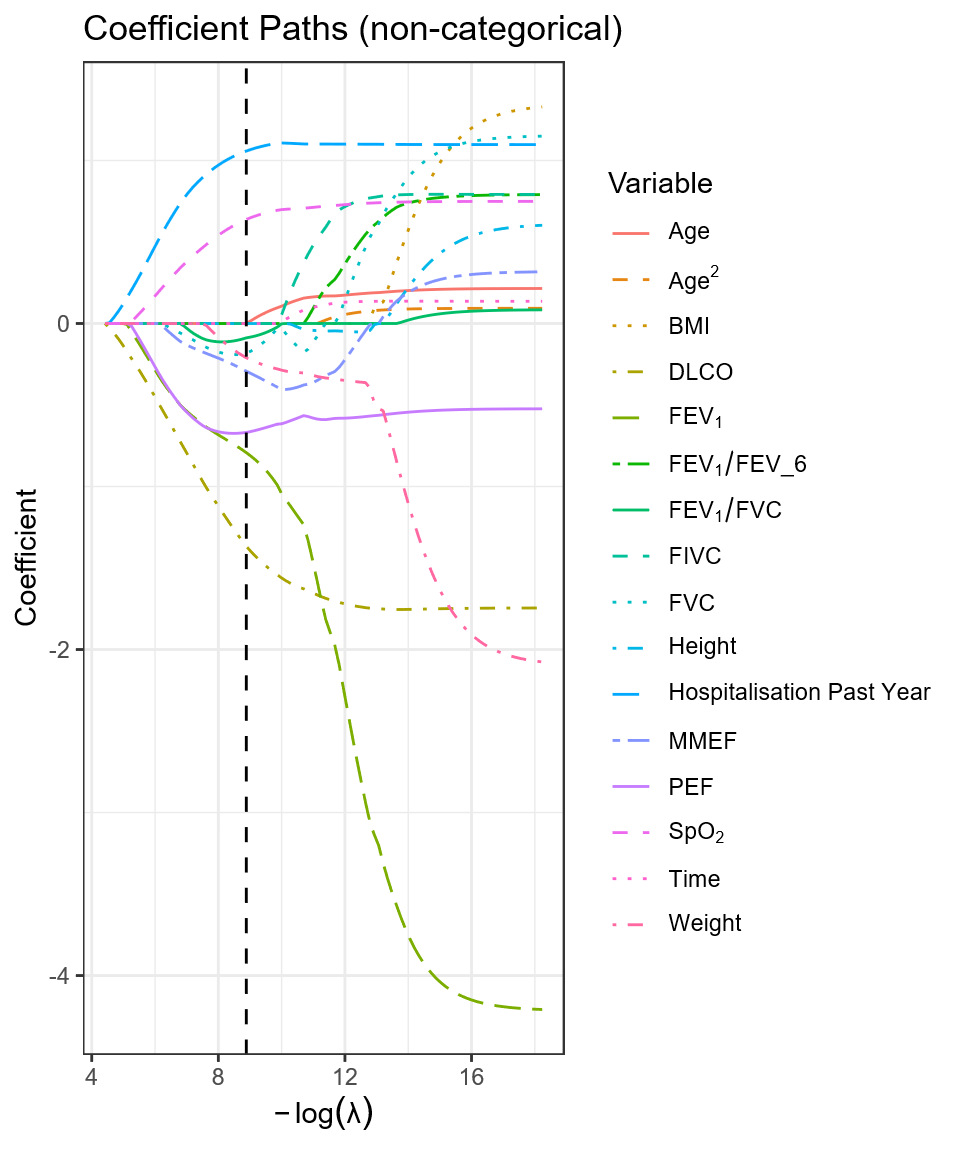 | 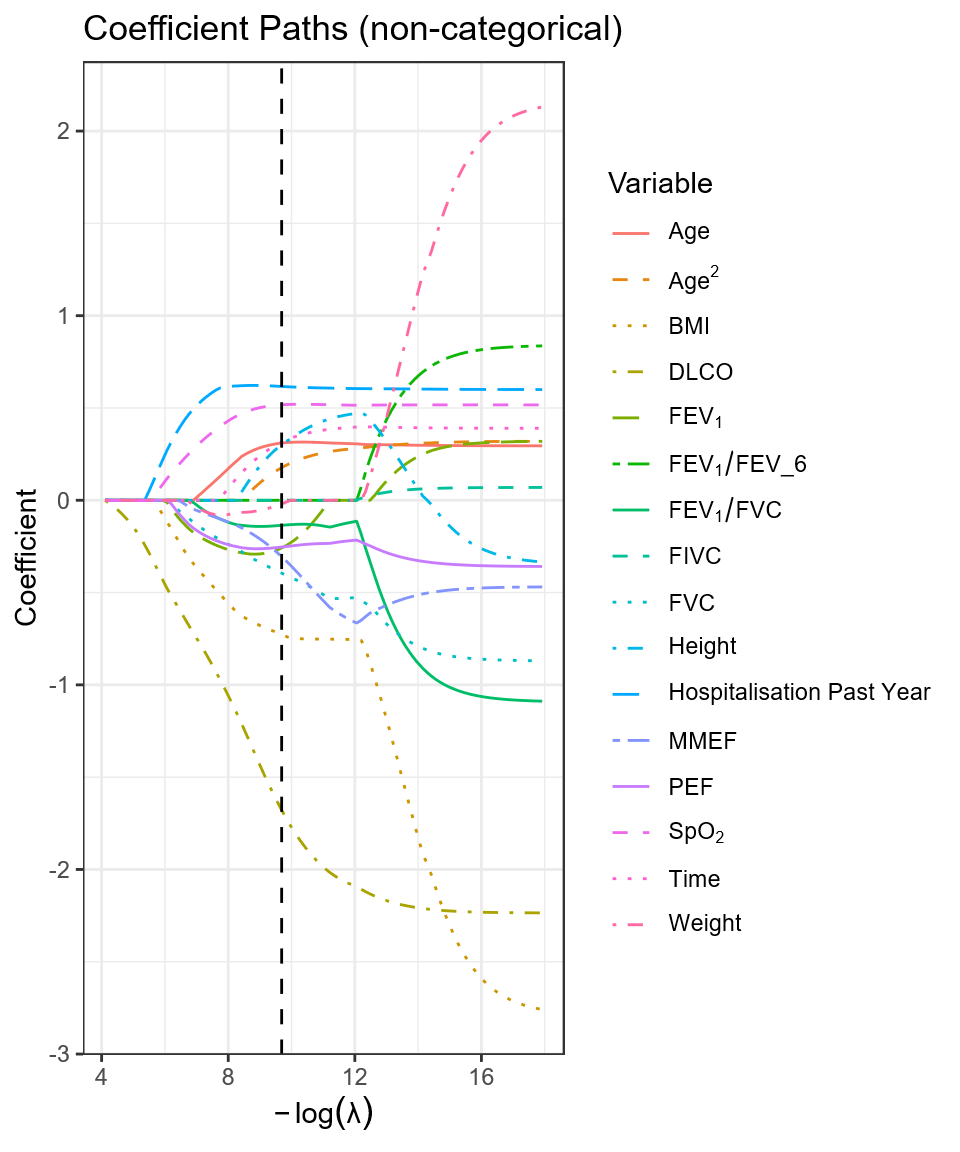 |
| 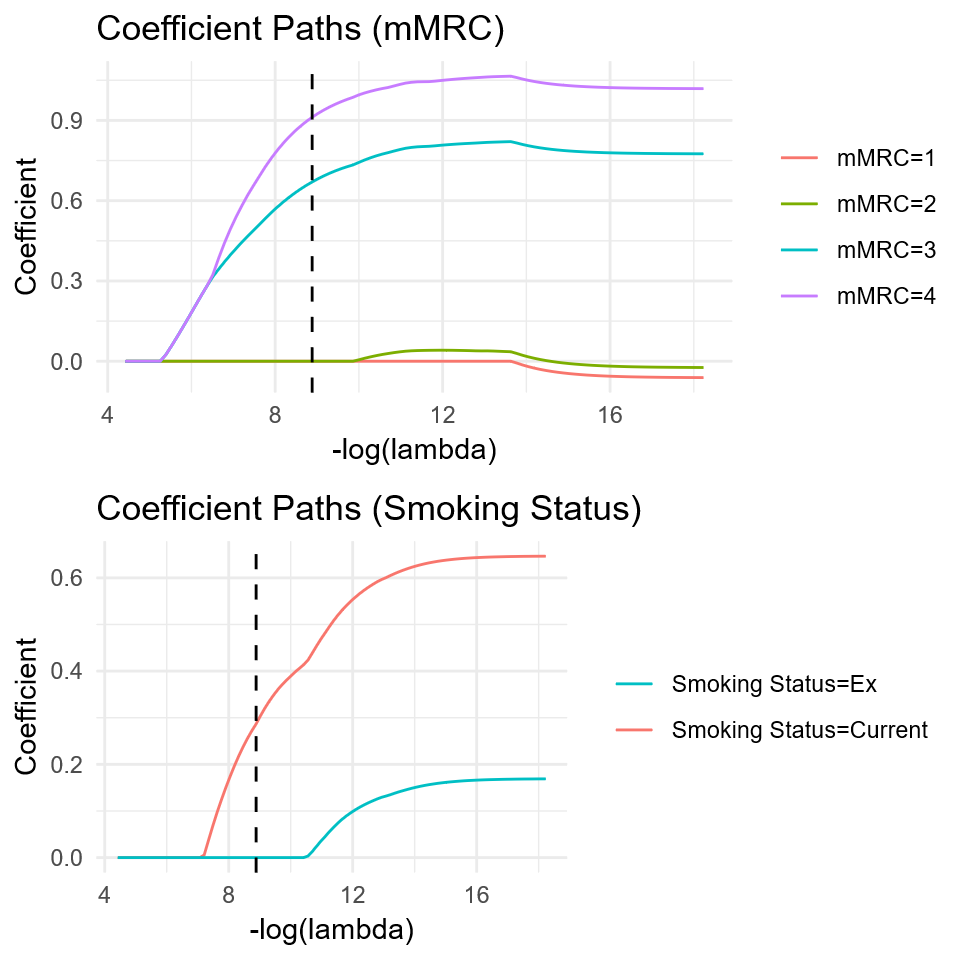 | 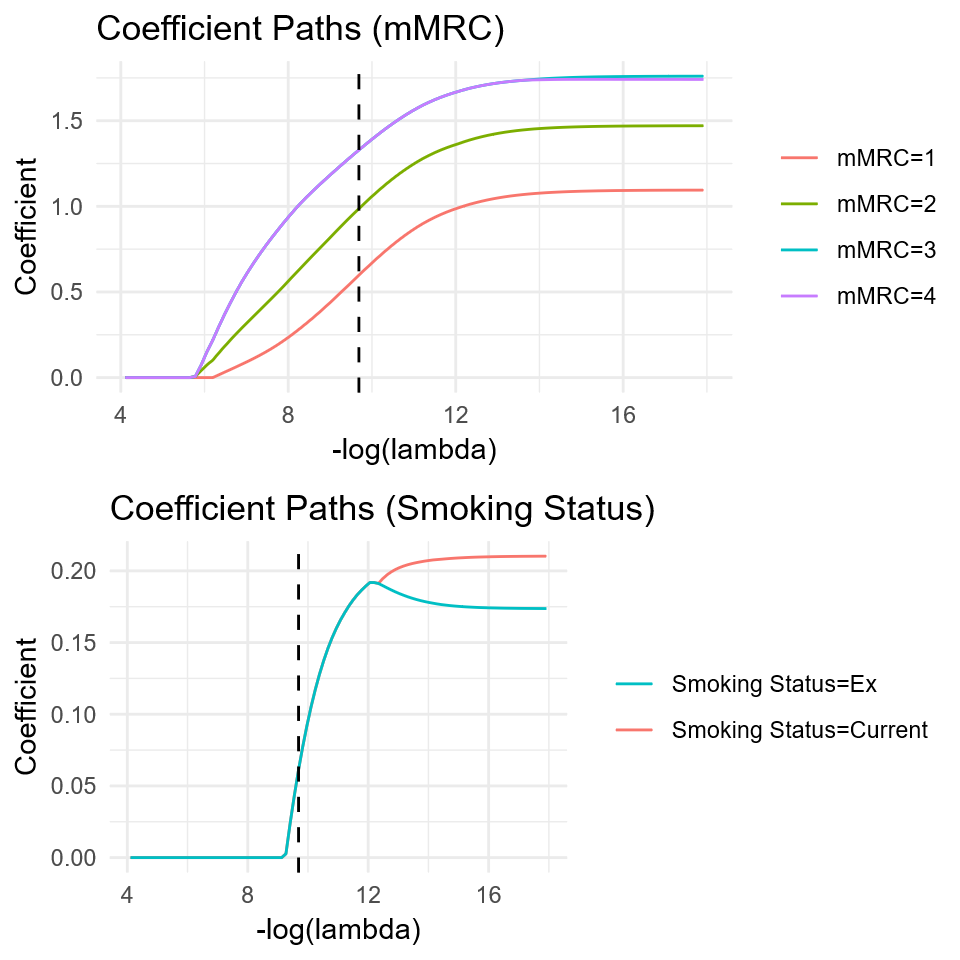 |

**Figure S11: Cross validated performances from penalised logistic models for mortality after 5 years.**

| **Female** | **Male** |
| --- | --- |
| 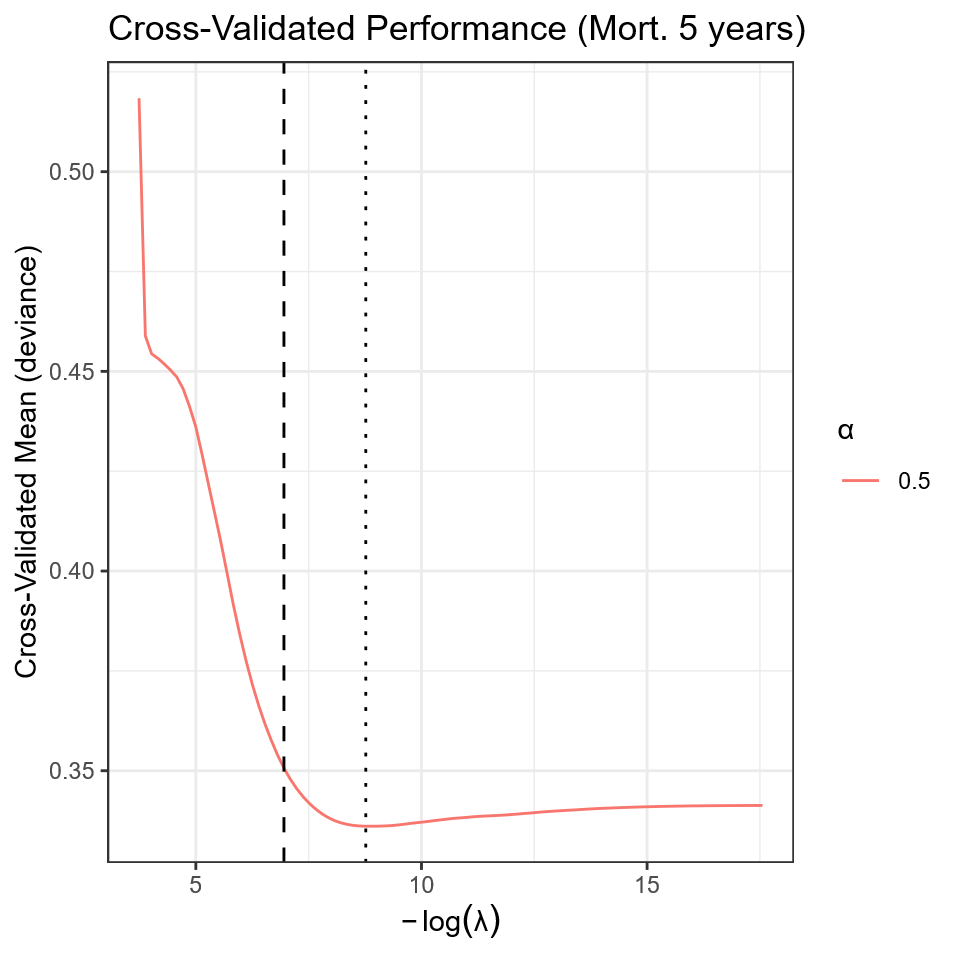 | 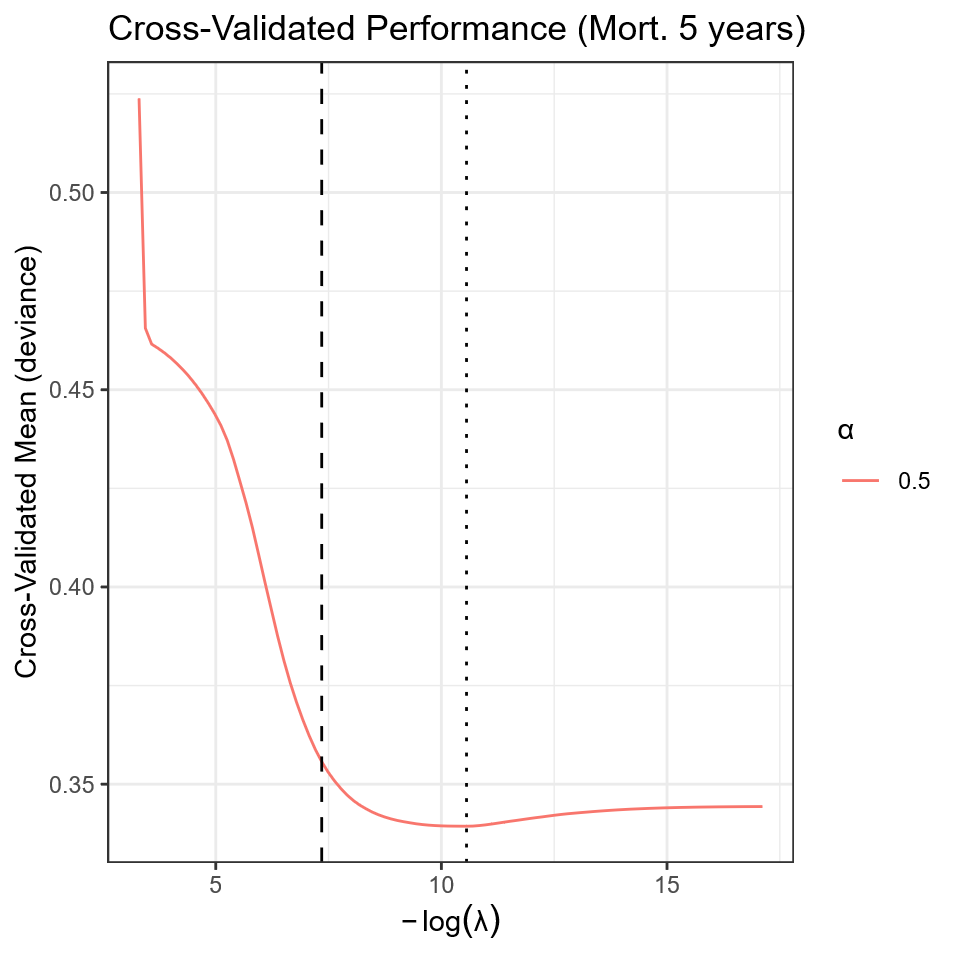 |

**Figure S12: Coefficient paths from penalised logistic models for mortality after 5 years.**

| **Female** | **Male** |
| --- | --- |
| 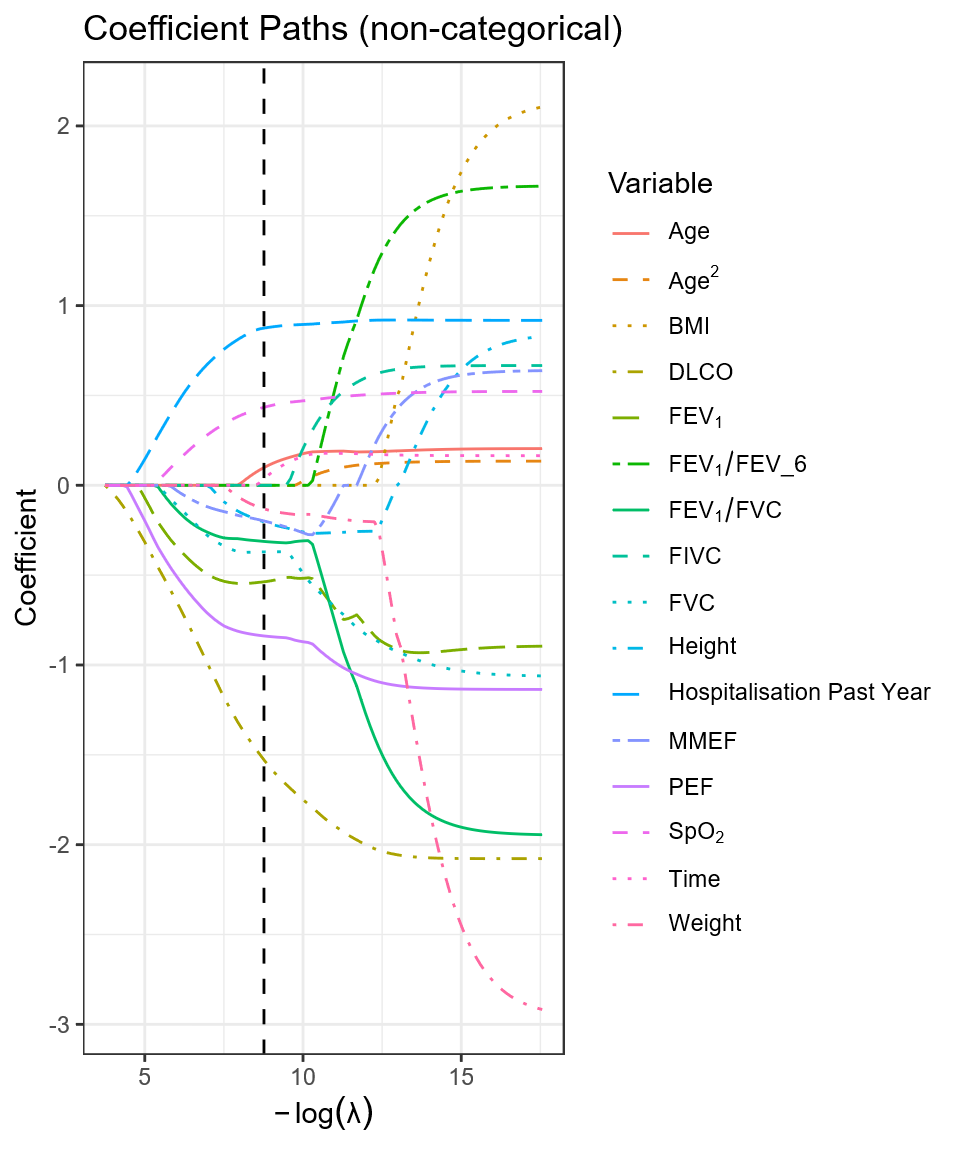 | 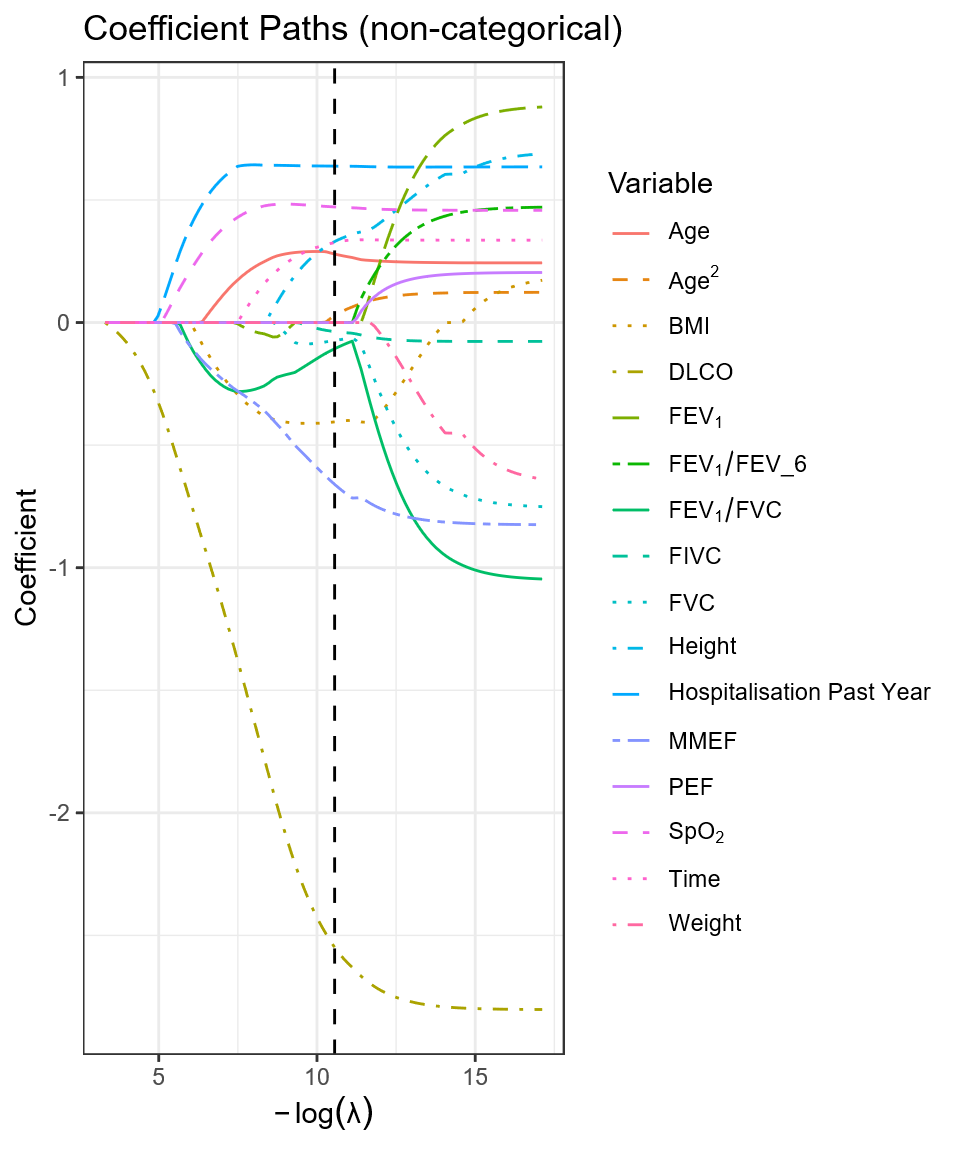 |
| 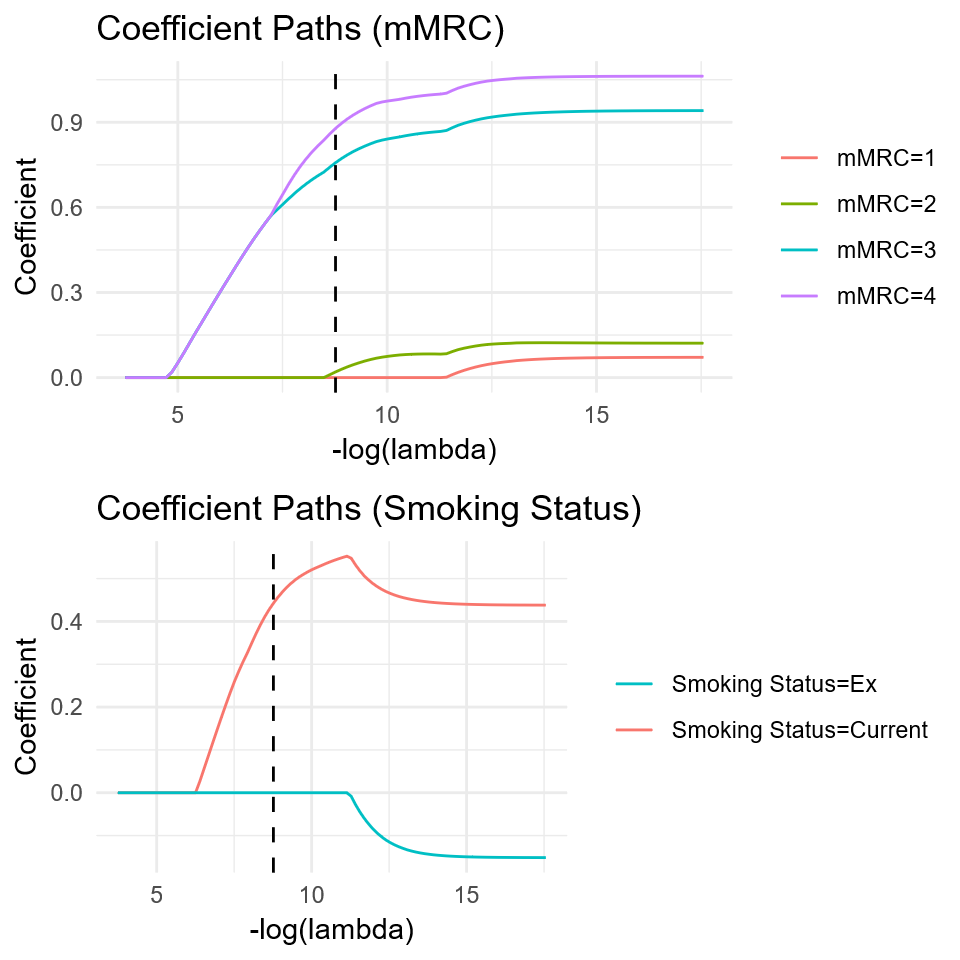 | 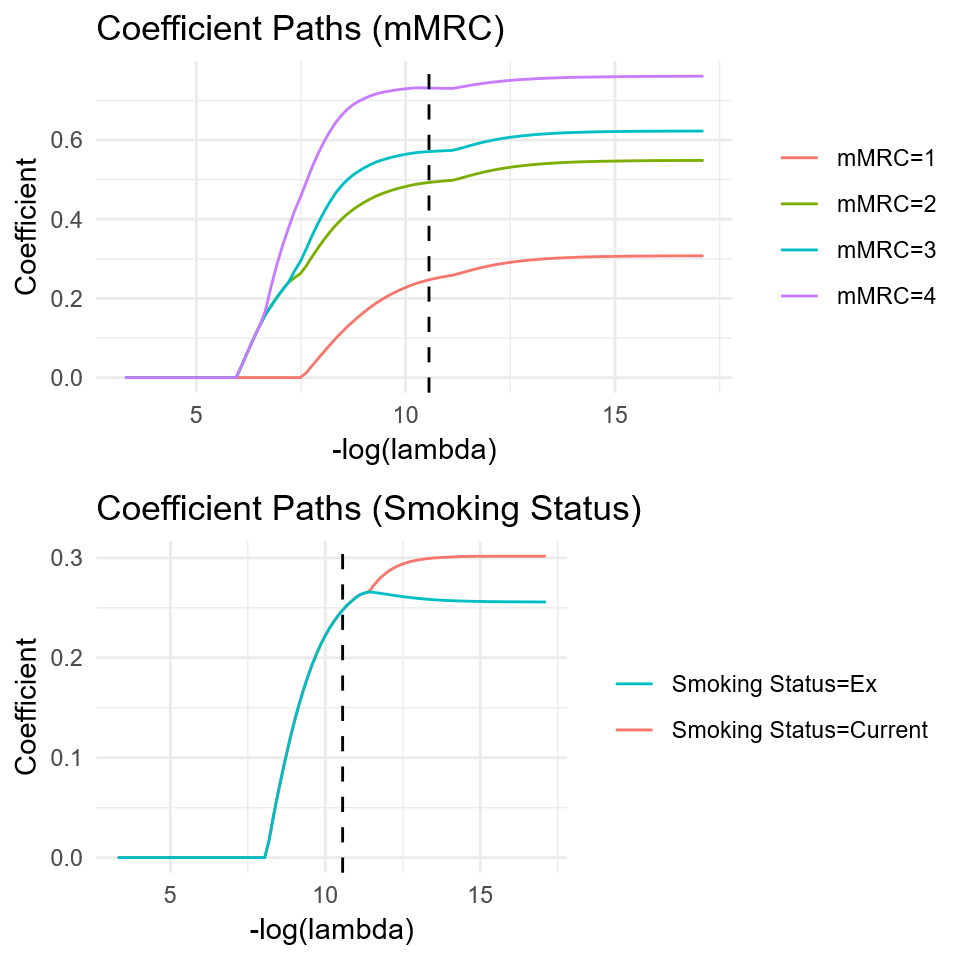 |

# Formulae for logistic regression models

**Expanded formulae for regression models**

The estimated risk for one of the nine outcomes modelled by GLMs with a binomial family and logit link functions is formally calculated with the below formula:

$$P\left( event \right)=\frac{1}{1+e^{-\boldsymbol{X}}}$$

Where $\boldsymbol{X}$ is the linear predictor for each outcome. In words, the linear predictor can be expressed as the sum of the differences between the population mean and the patient value, divided by twice the population standard deviation, for each predictor multiplied by the predictor coefficient. To convert from predictor coefficient to Odds ratio, the coefficient must be divided by twice the standard deviation and exponentiated. For example, the Odds ratio of FEV_1_ for 1-year hospitalisation in females is $\exp\left( -\frac{0.310}{2*0.740} \right)=0.811$. The linear predictors for each of the 10 outcomes are shown below.

Hospitalisation (1 year, female)

$$-2.350 + \left( 0.224 \right)*\left( \frac{Decades since 2006- 0.56}{2*0.30} \right)+ \left( -0.577 \right)*\left( \frac{DLCOc- 5.22}{2*2.02} \right)+ \left( -0.310 \right)*\left( \frac{FEV_{1} - 1.82}{2*0.74} \right)+ \left( -0.445 \right)*\left( \frac{\frac{FEV_{1}}{FEV_{6}}- 62.77}{2*10.12} \right)+ \left( -0.412 \right)*\left( \frac{MMEF - 0.76}{2*0.46} \right)+ \left( 0.239 \right)*\left( \frac{\ln\left( 101-SpO_{2} \right) - 1.52}{2*0.42} \right)+ \left( 0.897 \right)*\left( hospitalisation past year \right)+ 0.061*mMRC:1 + 0.192*mMRC:2 + 0.303*mMRC:3 + 0.303*mMRC:4 + 0.173*(smoking status):ex + 0.173*(smoking status):current$$

Hospitalisation (3 years, female)

$$-1.885 + \left( 0.234 \right)*\left( \frac{Decades since 2006- 0.56}{2*0.30} \right)+ \left( -0.802 \right)*\left( \frac{DLCOc- 5.22}{2*2.02} \right)+ \left( -0.275 \right)*\left( \frac{FEV_{1}- 1.82}{2*0.74} \right)+ \left( -0.445 \right)*\left( \frac{FEV_{1}/FEV_{6}- 62.77}{2*10.12} \right)+ \left( -0.454 \right)*\left( \frac{MMEF - 0.76}{2*0.46} \right)+ \left( 0.252 \right)*\left( \frac{\ln\left( 101-SpO_{2} \right) - 1.52}{2*0.42} \right)+ \left( 0.876 \right)*\left( hospitalisation past year \right)+ 0.116*mMRC:1 + 0.305*mMRC:2 + 0.385*mMRC:3 + 0.385*mMRC:4 + 0.142*(smoking status):ex + 0.142*(smoking status):current$$

Hospitalisation (5 years, female)

$$-2.005 + \left( -0.166 \right)*\left( \frac{\left( Age- 7.02 \right)^{2}}{2*1.05} \right)+ \left( 0.104 \right)*\left( \frac{BMI - 28.38}{2*6.27} \right)+ \left( 0.378 \right)*\left( \frac{Decades since 2006- 0.56}{2*0.30} \right)+ \left( -0.874 \right)*\left( \frac{DLCOc- 5.22}{2*2.02} \right)+ \left( -0.280 \right)*\left( \frac{FEV_{1}- 1.82}{2*0.74} \right)+ \left( -0.654 \right)*\left( \frac{FEV_{1}/FEV_{6}- 62.77}{2*10.12} \right)+ \left( -0.034 \right)*\left( \frac{FEV_{1}/FVC- 55.76}{2*12.19} \right)+ \left( -0.219 \right)*\left( \frac{MMEF - 0.76}{2*0.46} \right)+ \left( 0.285 \right)*\left( \frac{\ln\left( 101-SpO_{2} \right) - 1.52}{2*0.42} \right)+ \left( 1.053 \right)*\left( hospitalisation past year \right)+ 0.219*mMRC:1 + 0.586*mMRC:2 + 0.721*mMRC:3 + 0.721*mMRC:4 + 0.372*(smoking status):ex + 0.603*(smoking status):current$$

Mortality (3 years, female)

$$-4.521 + \left( -0.867 \right)*\left( \frac{DLCOc- 5.22}{2*2.02} \right)+ \left( -0.572 \right)*\left( \frac{FEV_{1}- 1.82}{2*0.74} \right)+ \left( -0.060 \right)*\left( \frac{FEV_{1}/FVC- 55.76}{2*12.19} \right)+ \left( -0.101 \right)*\left( \frac{FVC - 3.26}{2*1.05} \right)+ \left( -0.150 \right)*\left( \frac{MMEF - 0.76}{2*0.46} \right)+ \left( -0.581 \right)*\left( \frac{PEF - 5.59}{2*2.18} \right)+ \left( 0.417 \right)*\left( \frac{\ln\left( 101-SpO_{2} \right) - 1.52}{2*0.42} \right)+ \left( 0.839 \right)*\left( hospitalisation past year \right)+ 0.445*mMRC:3 + 0.582*mMRC:4 + 0.006*(smoking status):current$$

Mortality (5 years, female)

$$-4.044 + \left( -0.981 \right)*\left( \frac{DLCOc- 5.22}{2*2.02} \right)+ \left( -0.492 \right)*\left( \frac{FEV_{1}- 1.82}{2*0.74} \right)+ \left( -0.259 \right)*\left( \frac{FEV_{1}/FVC- 55.76}{2*12.19} \right)+ \left( -0.275 \right)*\left( \frac{FVC - 3.26}{2*1.05} \right)+ \left( -0.118 \right)*\left( \frac{MMEF - 0.76}{2*0.46} \right)+ \left( -0.707 \right)*\left( \frac{PEF - 5.59}{2*2.18} \right)+ \left( 0.277 \right)*\left( \frac{\ln\left( 101-SpO_{2} \right) - 1.52}{2*0.42} \right)+ \left( 0.670 \right)*\left( hospitalisation past year \right)+ 0.518*mMRC:3 + 0.518*mMRC:4 + 0.147*(smoking status):current$$

Hospitalisation (1 year, male)

$$-2.194 + \left( 0.132 \right)*\left( \frac{Decades since 2006- 0.56}{2*0.30} \right)+ \left( -0.377 \right)*\left( \frac{DLCOc- 5.22}{2*2.02} \right)+ \left( -0.405 \right)*\left( \frac{FEV_{1}/FEV_{6}- 62.77}{2*10.12} \right)+ \left( -0.244 \right)*\left( \frac{MMEF - 0.76}{2*0.46} \right)+ \left( -0.492 \right)*\left( \frac{PEF - 5.59}{2*2.18} \right)+ \left( 0.950 \right)*\left( hospitalisation past year \right)+ 0.228*mMRC:1 + 0.380*mMRC:2 + 0.380*mMRC:3 + 0.539*mMRC:4$$

Hospitalisation (3 years, male)

$$-1.745 + \left( 0.235 \right)*\left( \frac{Decades since 2006- 0.56}{2*0.30} \right)+ \left( -0.674 \right)*\left( \frac{DLCOc- 5.22}{2*2.02} \right)+ \left( -0.444 \right)*\left( \frac{FEV_{1}/FEV_{6}- 62.77}{2*10.12} \right)+ \left( -0.190 \right)*\left( \frac{FEV_{1}/FVC- 55.76}{2*12.19} \right)+ \left( -0.164 \right)*\left( \frac{MMEF - 0.76}{2*0.46} \right)+ \left( -0.412 \right)*\left( \frac{PEF - 5.59}{2*2.18} \right)+ \left( 0.199 \right)*\left( \frac{\ln\left( 101-SpO_{2} \right) - 1.52}{2*0.42} \right)+ \left( 1.021 \right)*\left( hospitalisation past year \right)+ 0.274*mMRC:1 + 0.317*mMRC:2 + 0.317*mMRC:3 + 0.317*mMRC:4 + 0.129*(smoking status):ex + 0.148*(smoking status):current$$

Hospitalisation (5 years, male)

$$-1.509 + \left( -0.011 \right)*\left( \frac{\left( Age- 7.02 \right)^{2}}{2*1.05} \right)+ \left( 0.299 \right)*\left( \frac{Decades since 2006- 0.56}{2*0.30} \right)+ \left( -0.504 \right)*\left( \frac{DLCOc- 5.22}{2*2.02} \right)+ \left( -0.381 \right)*\left( \frac{FEV_{1}/FEV_{6}- 62.77}{2*10.12} \right)+ \left( -0.386 \right)*\left( \frac{FEV_{1}/FVC- 55.76}{2*12.19} \right)+ \left( -0.265 \right)*\left( \frac{MMEF - 0.76}{2*0.46} \right)+ \left( -0.337 \right)*\left( \frac{PEF - 5.59}{2*2.18} \right)+ \left( 0.234 \right)*\left( \frac{\ln\left( 101-SpO_{2} \right) - 1.52}{2*0.42} \right)+ \left( 0.974 \right)*\left( hospitalisation past year \right)+ 0.275*mMRC:1 + 0.275*mMRC:2 + 0.275*mMRC:3 + 0.275*mMRC:4 + 0.199*(smoking status):ex + 0.354*(smoking status):current$$

Mortality (3 year, male)

$$-4.010 + \left( 0.085 \right)*\left( \frac{Age - 7.02}{2*1.05} \right)+ \left( -0.455 \right)*\left( \frac{BMI - 28.38}{2*6.27} \right)+ \left( -0.880 \right)*\left( \frac{DLCOc- 5.22}{2*2.02} \right)+ \left( -0.231 \right)*\left( \frac{FEV_{1}- 1.82}{2*0.74} \right)+ \left( -0.077 \right)*\left( \frac{FEV_{1}/FVC- 55.76}{2*12.19} \right)+ \left( -0.184 \right)*\left( \frac{FVC - 3.26}{2*1.05} \right)+ \left( -0.083 \right)*\left( \frac{MMEF - 0.76}{2*0.46} \right)+ \left( -0.207 \right)*\left( \frac{PEF - 5.59}{2*2.18} \right)+ \left( 0.363 \right)*\left( \frac{\ln\left( 101-SpO_{2} \right) - 1.52}{2*0.42} \right)+ \left( -0.072 \right)*\left( \frac{Weight - 79.36}{2*19.64} \right)+ \left( 0.579 \right)*\left( hospitalisation past year \right)+ 0.148*mMRC:1 + 0.426*mMRC:2 + 0.767*mMRC:3 + 0.767*mMRC:4$$

Mortality (5 years, male)

$$-3.213 + \left( 0.156 \right)*\left( \frac{Age - 7.02}{2*1.05} \right)+ \left( -0.273 \right)*\left( \frac{BMI - 28.38}{2*6.27} \right)+ \left( -1.311 \right)*\left( \frac{DLCOc- 5.22}{2*2.02} \right)+ \left( -0.278 \right)*\left( \frac{FEV_{1}/FVC- 55.76}{2*12.19} \right)+ \left( -0.266 \right)*\left( \frac{MMEF - 0.76}{2*0.46} \right)+ \left( 0.417 \right)*\left( \frac{\ln\left( 101-SpO_{2} \right) - 1.52}{2*`0.42} \right)+ \left( 0.625 \right)*\left( hospitalisation past year \right)+ 0.253*mMRC:2 + 0.270*mMRC:3 + 0.421*mMRC:4$$
